# Supplementary material for: Molecular landscape and subtype-specific therapeutic response of nasopharyngeal carcinoma revealed by integrative pharmacogenomics
Source: Nat Commun. 2021 May 24;12:3046. doi: 10.1038/s41467-021-23379-3 (PMC8144567; doi:10.1038/s41467-021-23379-3)
Supplement: Supplementary file 3 — Supplementary information [file 41467_2021_23379_MOESM3_ESM.pdf]

Supplementary Information

Molecular Landscape and Subtype-Specific Therapeutic Response of Nasopharyngeal Carcinoma Revealed by Integrative Pharmacogenomics

Ding et al.

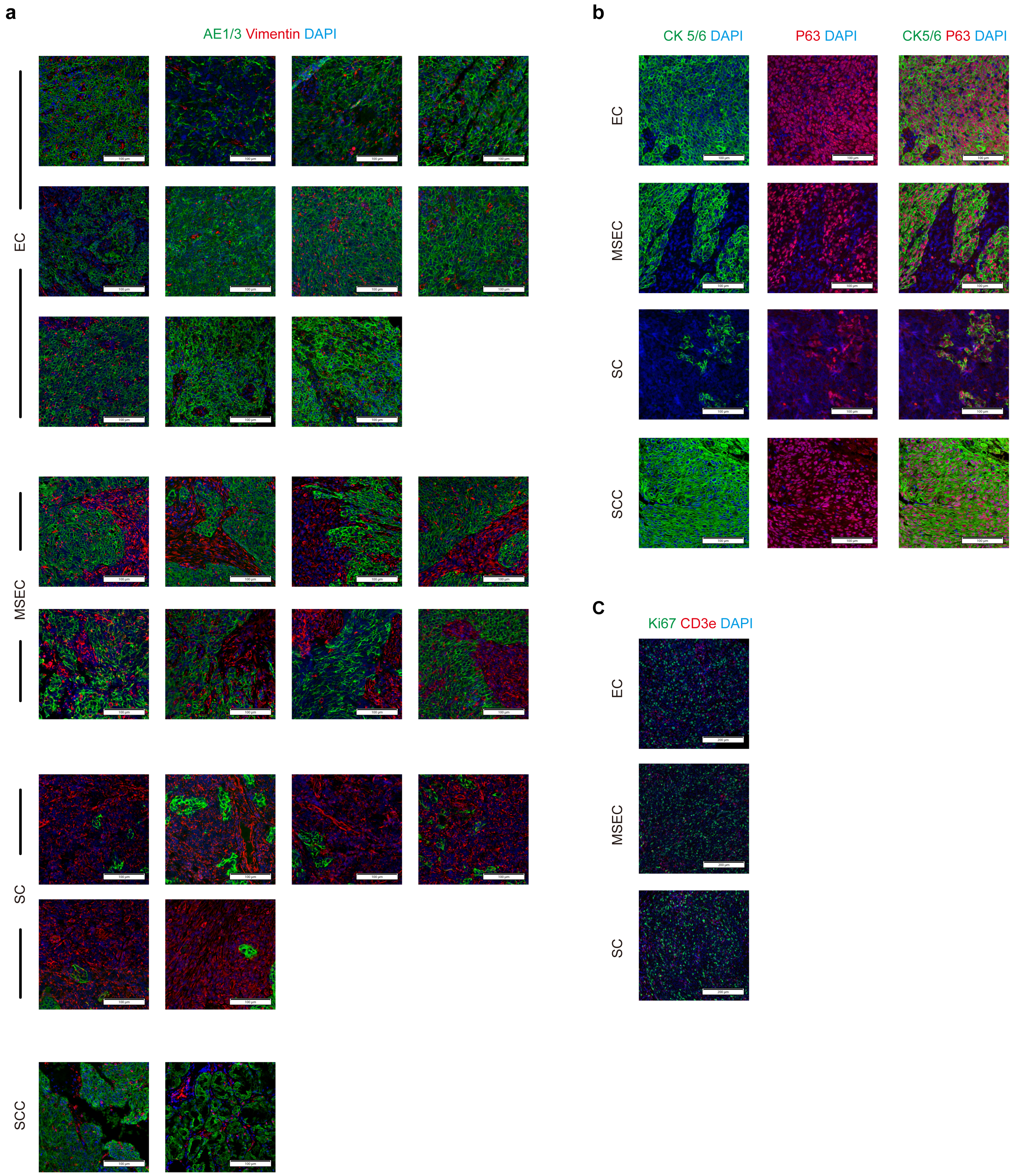

Supplementary Figure 1 Immunohistochemical staining of NPC markers. a Immunohistochemical staining of epithelial cell marker AE1/3 and sarcomatoid cell marker vimentin in four subtypes of NPC. They generally displayed subtype-specific difference. Scale bar, 100 μm. **b** Immunohistochemical staining of epithelial cell markers CK5/6 and P63. They were strongly expressed in EC subtype rather than in SC subtype. Scale bar, 100 μm. The immunohistochemical images (a, b) are representatives of 31 tumors consist of 12 EC subtype, 7 SC subtype, 9 MSEC subtype and 3 SCC subtype tumors. **c** Immunohistochemical staining of proliferating cell maker Ki67 and T cell marker CD3e in EC, MSEC and SC subtypes of NPC. There was no significant difference among subtypes. Scale bar, 200 μm.

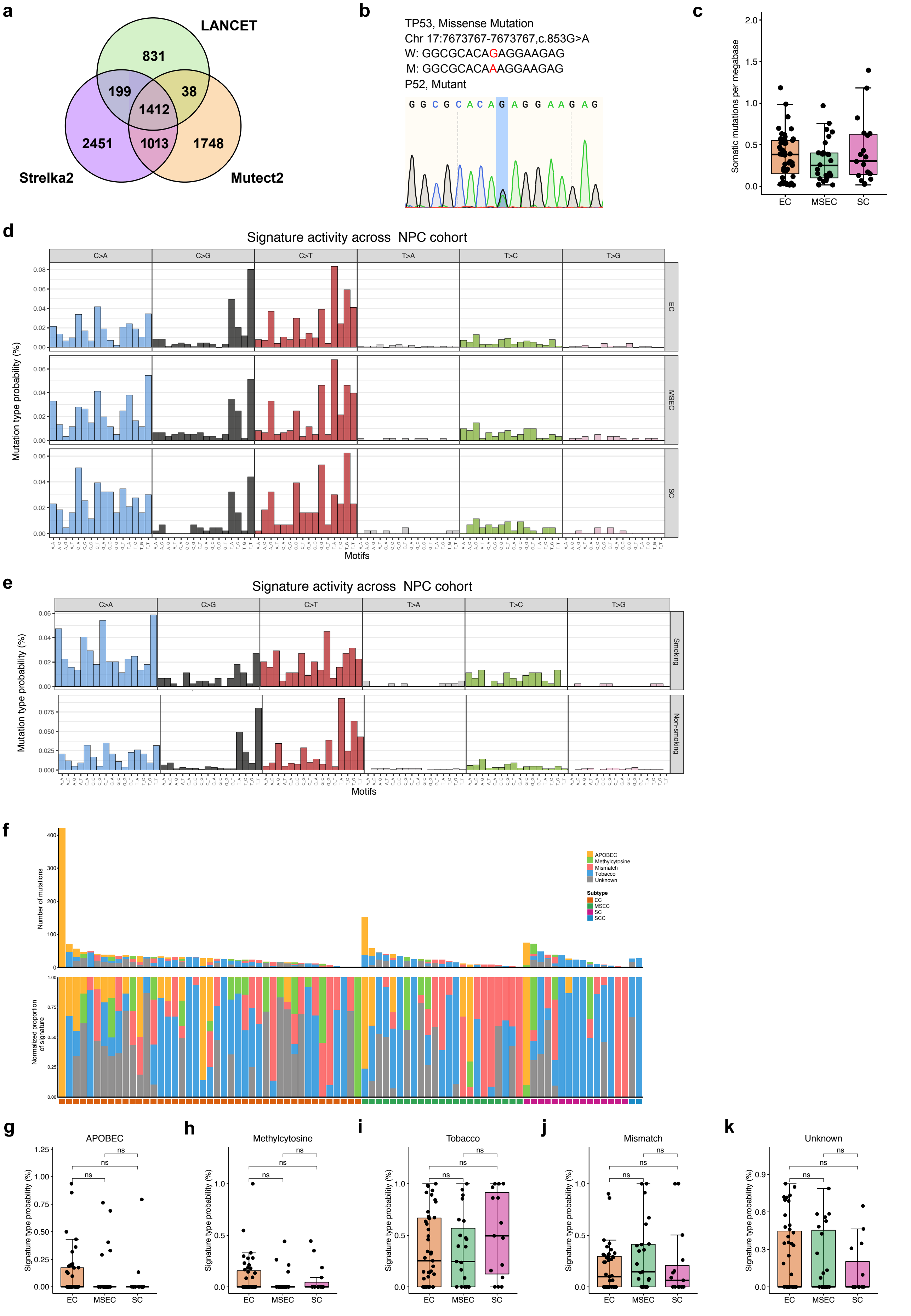
a
LANCET
831
199
38
1412
2451
1013
1748
Strelka2
Mutect2
b
TP53, Missense Mutation
Chr 17:7673767-7673767,c.853G>A
W: GGCGCACAGAGGAAGAG
M: GGCGCACAAAGGAAGAG
P52, Mutant
G G C G C A C A G A G G A A G A G
c
Somatic mutations per megabase
EC
MSEC
SC
d
Signature activity across NPC cohort
C>A
C>G
C>T
T>A
T>C
T>G
EC
MSEC
SC
Mutation type probability (%)
Motifs
e
Signature activity across NPC cohort
Smoking
Non-smoking
Mutation type probability (%)
Motifs
f
Number of mutations
Normalized proportion of signature
APOBEC
Methylcytosine
Mismatch
Tobacco
Unknown
Subtype
EC
MSEC
SC
SCC
g
APOBEC
h
Methylcytosine
i
Tobacco
j
Mismatch
k
Unknown
Signature type probability (%)
ns
EC
MSEC
SC

Supplementary Figure 2 COSMIC mutational signature of NPC. a Mutations called by MuTect2, Strelka2 and Strelka2 with two-two overlap (n=88 tumors). **b** Sanger sequencing validation on key candidate mutation, *TP53*. More validation results can be found in Supplementary Data 4. **c** Comparison of somatic mutation rates among NPC subtypes. Box plots represent median levels, first and third percentiles. Whiskers indicate 1.5 × inter-quartile range (IQR) extending from the hinges. Statistical significance was calculated using two-sided *t*-test, with no significant difference found among subtypes, $p > 0.05$. Data points are displayed as dots. 46 EC, 23 MSEC and 16 SC tumors were analyzed. **d** Comparison of overall mutational signatures among NPC subtypes. EC, MSEC and SC displayed similar mutational signatures. **e** Comparison of overall mutational signatures between smoking and non-smoking patients, C>A transition is associated with smoking status in NPC. **f-k** Distributions of top COSMIC NPC mutational signatures of each subtype. Box plots (g-k) represent median levels, first and third percentiles. Whiskers indicate 1.5 × inter-quartile range (IQR) extending from the hinges. Data points (g-k) are displayed as dots. Statistical significance (g-k) was calculated using two-sided *t*-test, with no significant difference found among subtypes, $p > 0.05$. 46 EC, 23 MSEC and 16 SC tumors were analyzed.

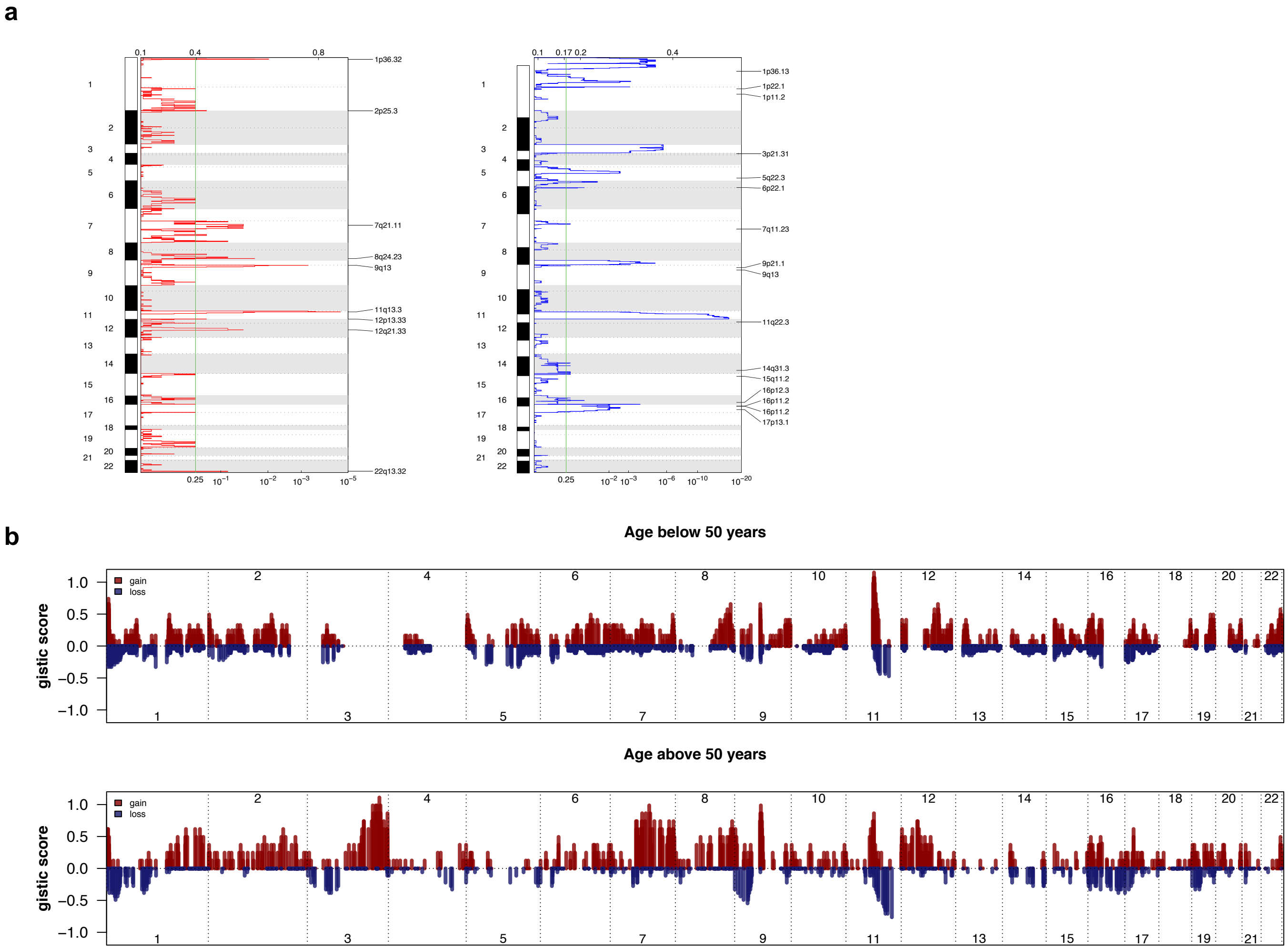

Supplementary Figure 3 Copy number alterations (CNVs) in NPC. a Chromosomal arm-level CNVs detected by GISTIC 2.0. Regions of recurrent amplifications (left, red) and deletions (right, blue) (n=99 tumors). **b** Chromosomal arm-level amplification (red) and deletion (blue) events identified in different age groups (n=99 tumors).

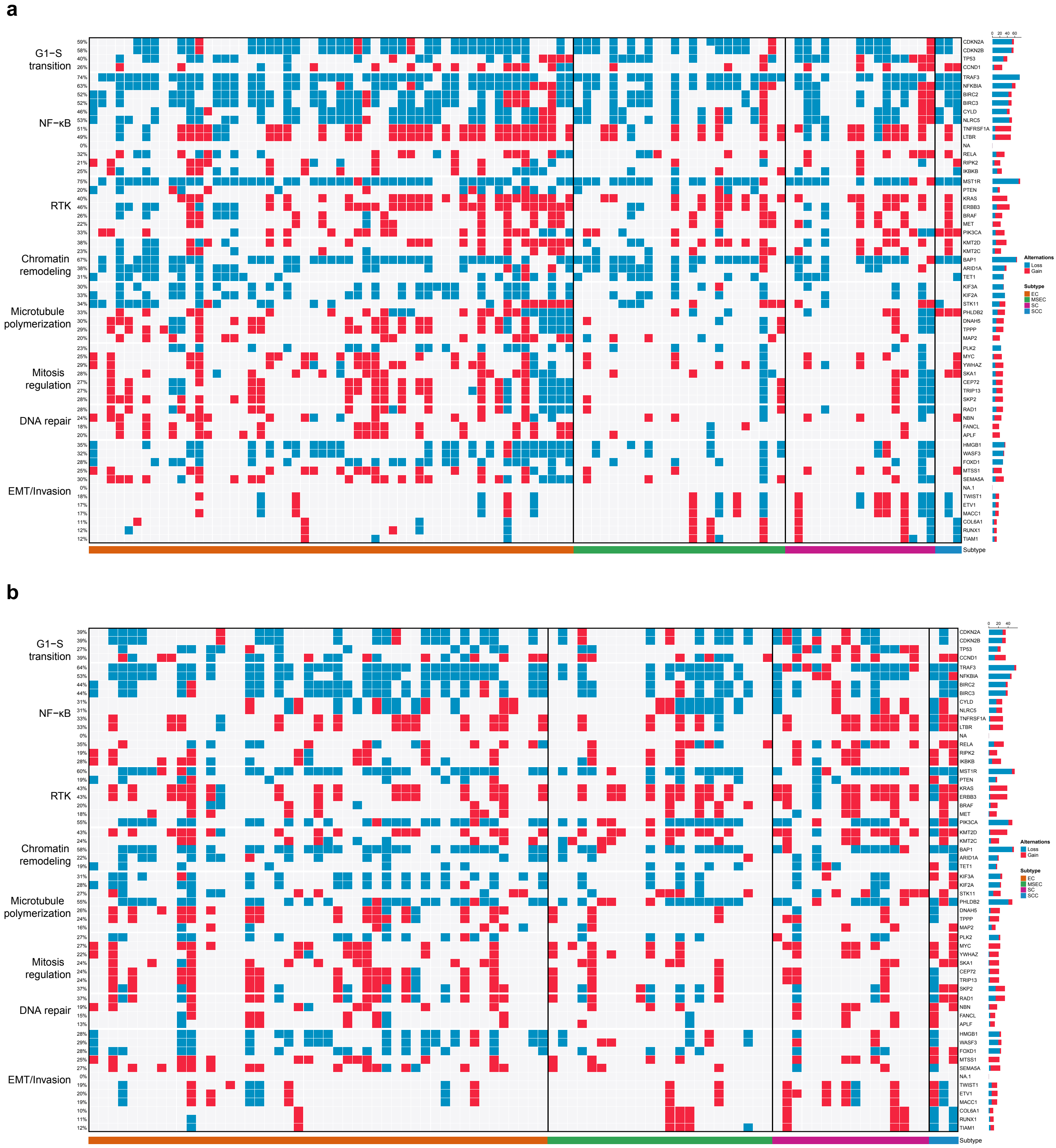

Supplementary Figure 4 Top frequent CNVs revealed common and subtype-specific driver mutational pathways of NPC. a Top frequent CNVs and associated pathways called by CNVkit program. **b** Top frequent CNVs and associated pathways called by Sequenza program.

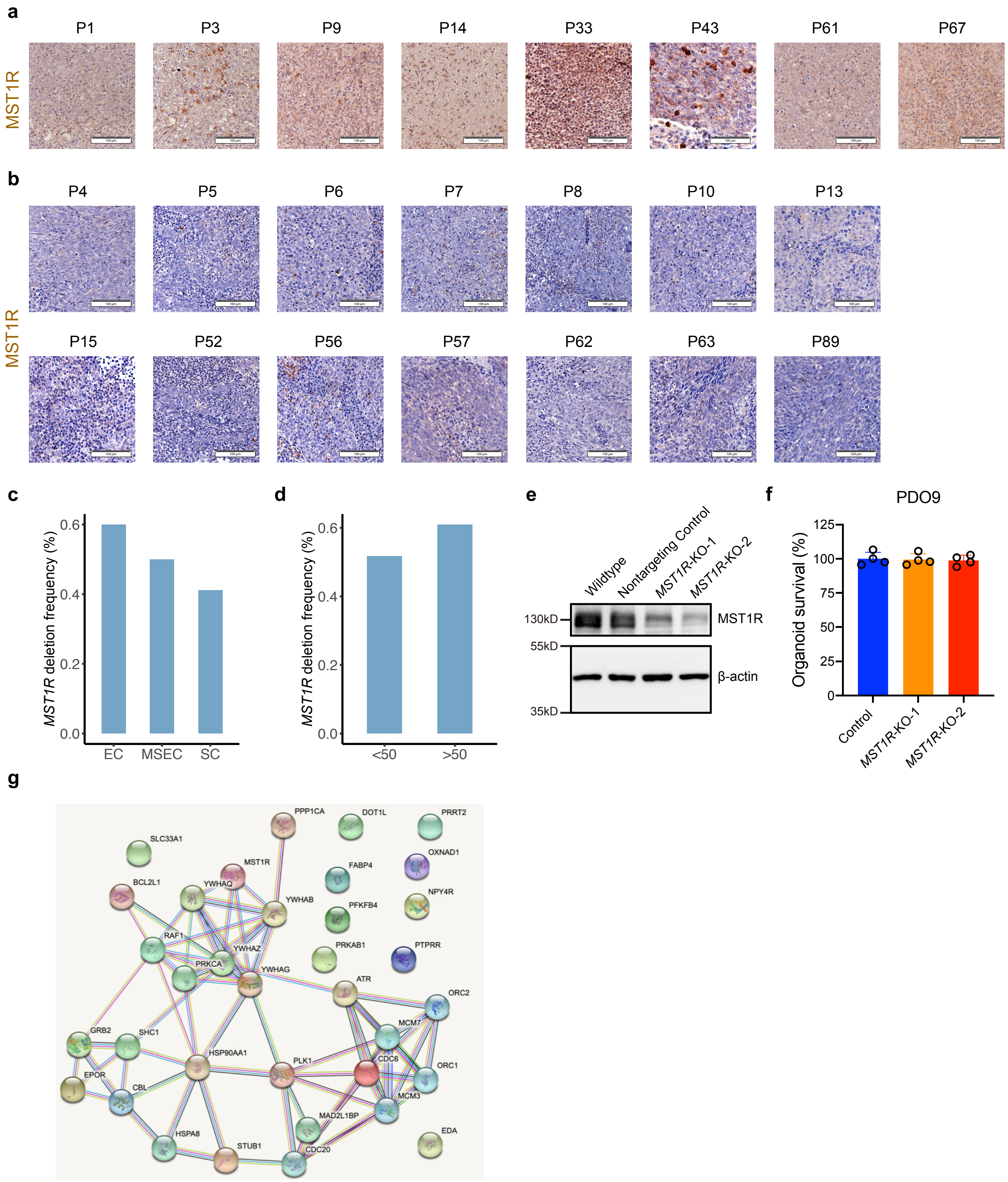

Supplementary Figure 5 *MST1R* is frequently deleted in NPC. a Immunohistochemical staining of MST1R in wildtype tumors (n=8 tumors). Scale bar, 100 µm. **b** Immunohistochemical staining of MST1R in NPC tumors with MST1R loss (n=14 tumors). Scale bar, 100 µm. **c** *MST1R* deletion frequencies among NPC subtypes. **d** *MST1R* deletion frequencies between different age groups. **e** Western blot validation on *MST1R* knockout (KO) in PDO9. Blots are representatives of two independent repeats. **f** *MST1R* knockout (KO) didn't affect organoid growth of wildtype PDO9. Data bars represent mean % viability relative to control ± SEM, for n = 4 biological replicates. Data points are displayed as dots. Graphs are representatives of 3 independent experiments. Statistical significance was calculated using two-sided *t*-test for comparing difference between nontargeting control and gene knockout groups, with no significant difference found, $p > 0.05$. **g** Protein-protein interaction network analysis indicated that 14-3-3 is the top interacting protein with MST1R.

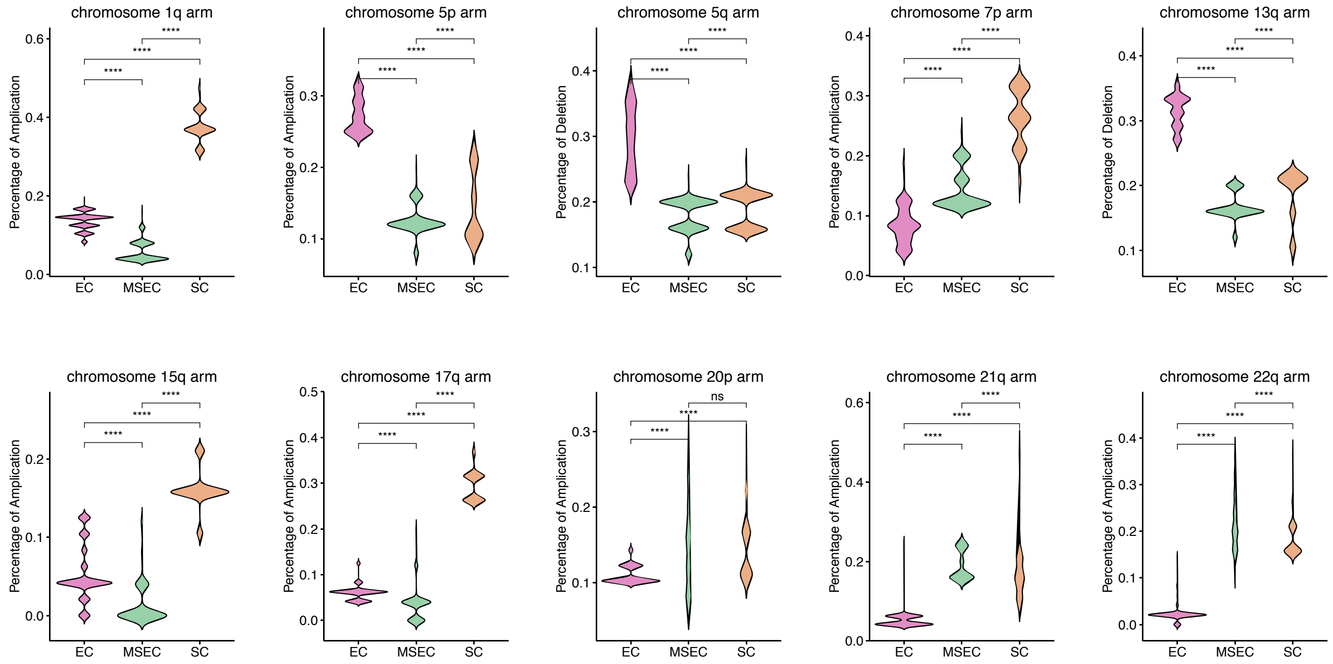

Supplementary Figure 6 Subtype-specific chromosomal gains and deletions. The distributions of chromosomal amplification of Chr. 1q, 5p, 7p, 15q, 17q, 20q, 21q and 22q, as well as deletion in Chr. 5q and 13q among NPC subtypes. Violin plots show the kernel probability density of the data at different values. Statistical significance was calculated using two-sided *t*-test, with ns p value > 0.05, **** $p \leq 0.0001$. 55 EC, 24 MSEC and 17 SC tumors were analyzed.

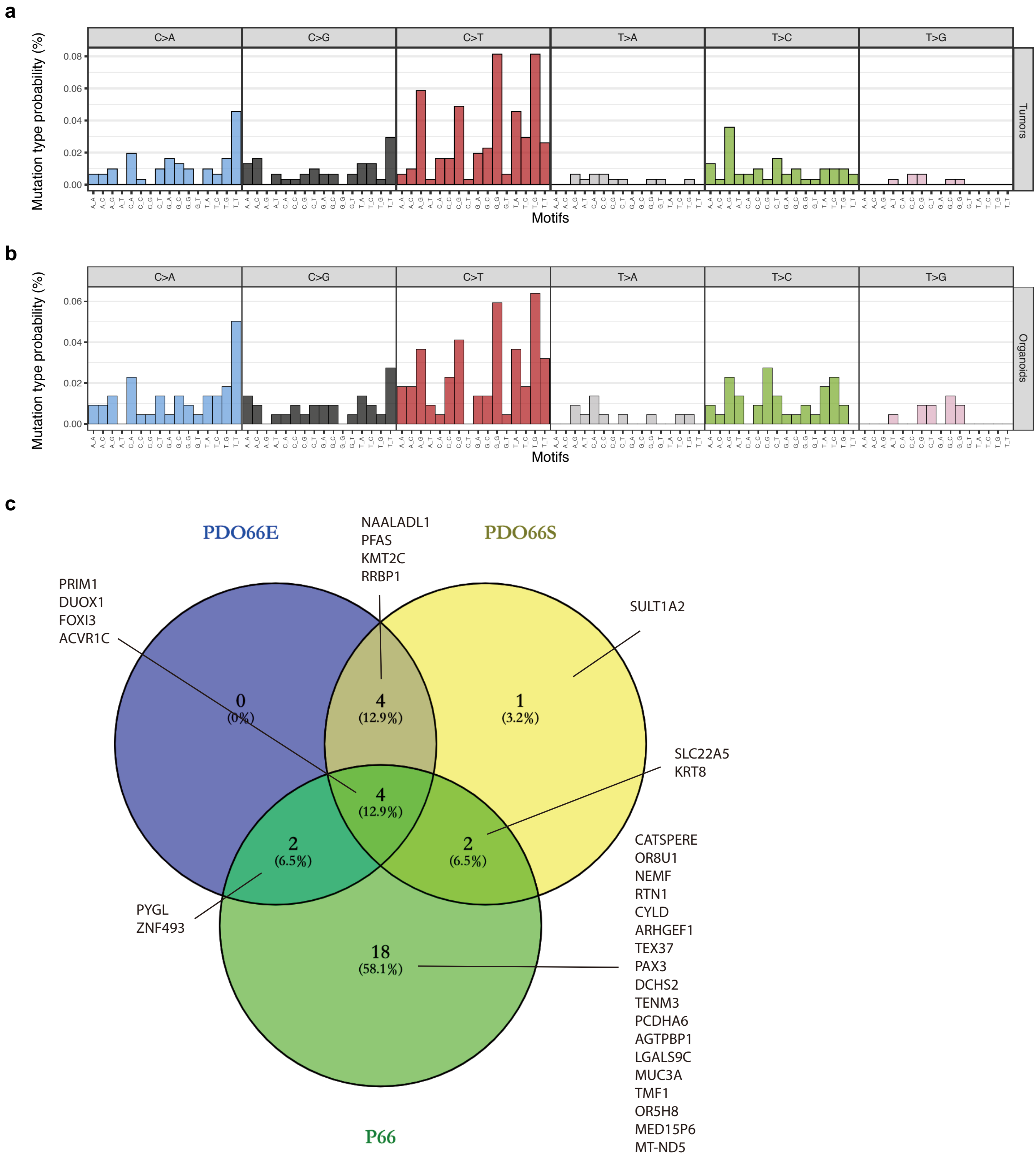

Supplementary Figure 7 Genomic comparison between paired tumors and PDOs. a, b Comparison of mutational signatures between paired tumor and PDOs. Consistent mutational signature profiles of parental tumors (a) were maintained in PDOs (b). **c** Somatic mutations identified from P66, PDO66E and PDO66S. Both common and distinct mutations were observed between PDOs originated from same MSEC tumor.

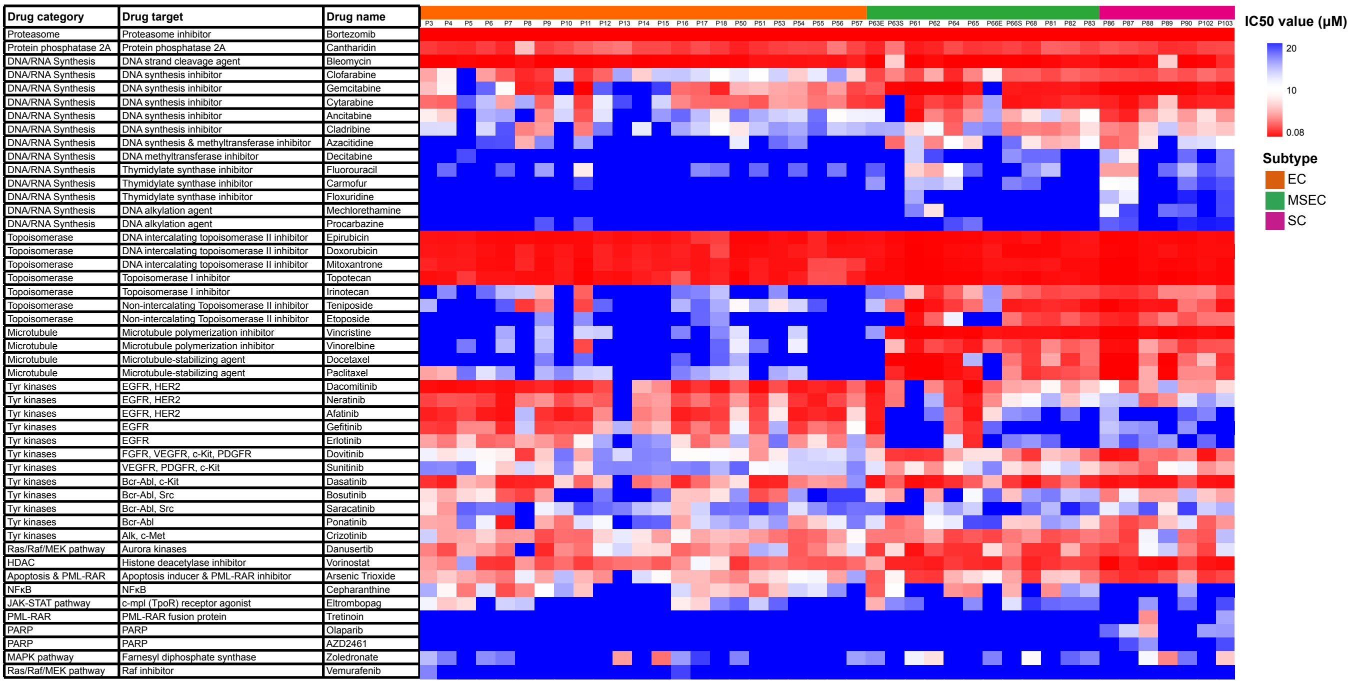

Supplementary Figure 8 Drug screening on patient-derived organoids (PDOs). Heatmap showing IC50 values of 48 drugs screening on 42 NPC PDOs.

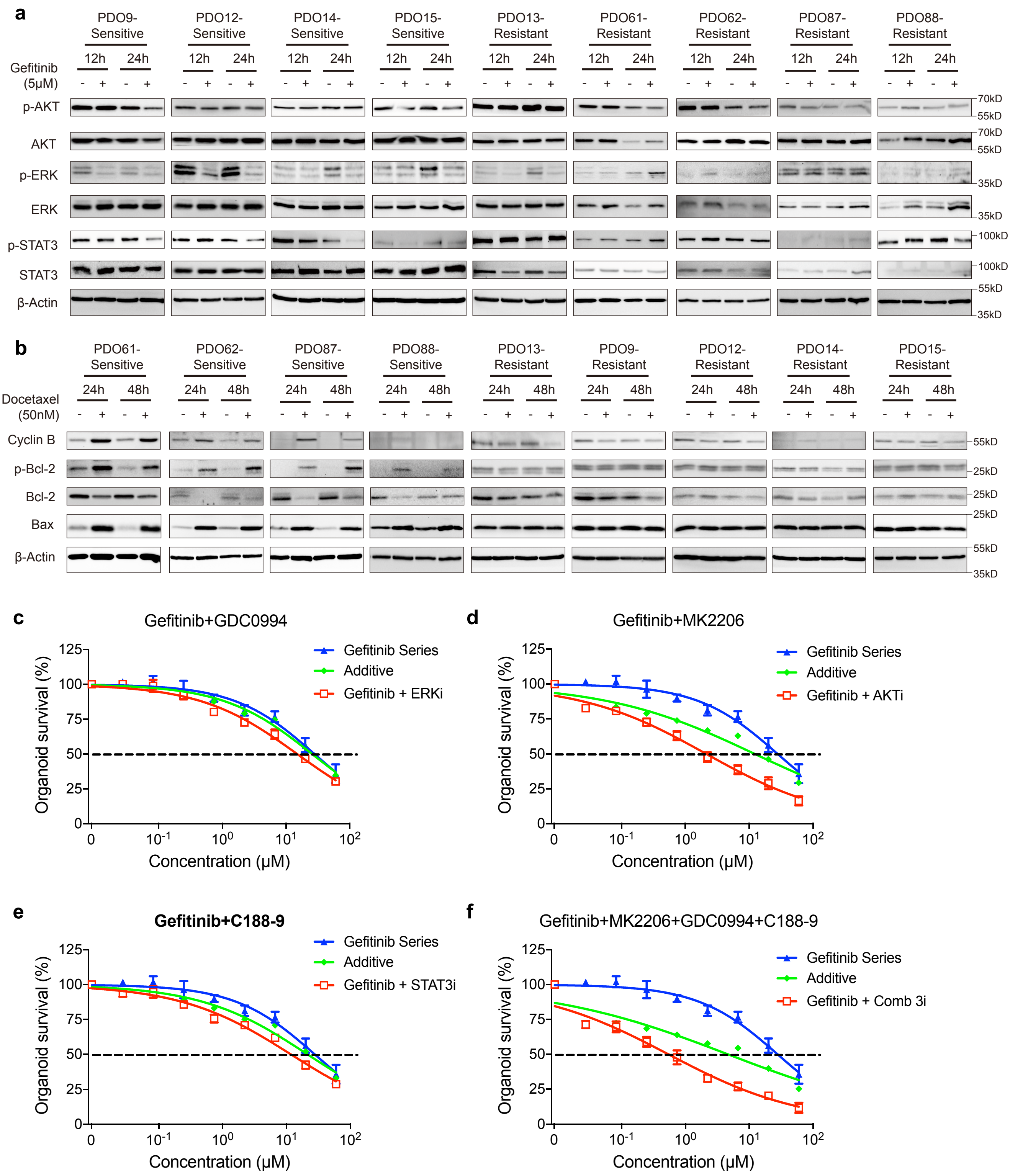

Supplementary Figure 9 Western blot analysis and combinational treatment on PDOs. **a** Expression changes of EGFR downstream proteins in PDOs when treated with gefitinib. **b** Expression changes of mitotic checkpoint and apoptotic proteins in PDOs when treated with docetaxel. Blots (a, b) are representatives of two independent repeats. **c** Combinational treatment of gefitinib and ERK inhibitor GDC0994 on PDO9. The synergistic effect was observed. **d** Combinational treatment of gefitinib and AKT inhibitor MK2206 on PDO9. Significantly synergistic effect was observed. **e** Combinational treatment of gefitinib and STAT3 inhibitor C188-9 on PDO9. The synergistic effect was observed. **f** Combinational treatment of gefitinib, GDC0994, MK2206 and C188-9 on PDO9. Significantly synergistic effect was observed. The single treatment curves (c-f) are presented with blue color; the combinational treatment curves (c-f) are labelled with red color; and the additive curves (c-f) are marked with green color. The additive curves (c-f) represent the estimated combinational treatment effect being equal to the sum of their separate effects. If the combinational treatment curve (red) is lower than the additive curve (green), the synergistic effect is demonstrated. Data points (c-f) represent mean % viability relative to control ± SEM for n = 3 biological replicates. Graphs (c-f) are representatives of 3 independent experiments.

a

Subtype	PDO		Dacomitinib	Neratinib	Afatinib	Gefitinib	Erlotinib	Average fold changes
EC	P4	IC50 (drug alone)	0.60	0.94	1.28	2.49	1.98	
		IC50 (drug + IR)	0.13	0.15	0.20	0.26	0.23	
		Fold changes	4.70	6.48	6.34	9.43	8.52	7.09
	P9	IC50 (drug alone)	0.63	0.70	0.90	1.06	1.24	
		IC50 (drug + IR)	0.14	0.15	0.15	0.15	0.17	
		Fold changes	4.56	4.68	5.93	6.94	7.25	5.87
	P11	IC50 (drug alone)	0.70	0.92	1.39	1.63	1.36	
		IC50 (drug + IR)	0.23	0.16	0.16	0.23	0.23	
		Fold changes	3.05	5.63	8.45	7.02	5.92	6.01
	P12	IC50 (drug alone)	0.58	1.00	1.43	2.27	2.38	
		IC50 (drug + IR)	0.08	0.14	0.22	0.23	0.23	
		Fold changes	6.86	7.36	6.60	9.87	10.15	8.17
MSEC	P62	IC50 (drug alone)	1.31	5.41	11.54	>20.00	>20.00	
		IC50 (drug + IR)	0.84	2.13	5.67	18.69	~20.00	
		Fold changes	1.57	2.54	2.03	1.07	1.00	1.64
	P66	IC50 (drug alone)	1.16	5.00	12.61	>20.00	>20.00	
		IC50 (drug + IR)	0.50	1.78	6.04	~20.00	~20.00	
		Fold changes	2.31	2.81	2.09	1.00	1.00	1.84
SC	P86	IC50 (drug alone)	1.54	6.06	13.66	>20.00	~20.00	
		IC50 (drug + IR)	0.57	1.90	8.10	8.75	7.35	
		Fold changes	2.72	3.18	1.69	2.28	2.72	2.52
	P88	IC50 (drug alone)	1.41	5.88	12.24	>20.00	>20.00	
		IC50 (drug + IR)	0.56	2.34	5.81	~20.00	~20.00	
		Fold changes	2.52	2.51	2.11	1.00	1.00	1.83

Subtype
- EC
- MSEC
- SC

Fold changes
- >4
- <3

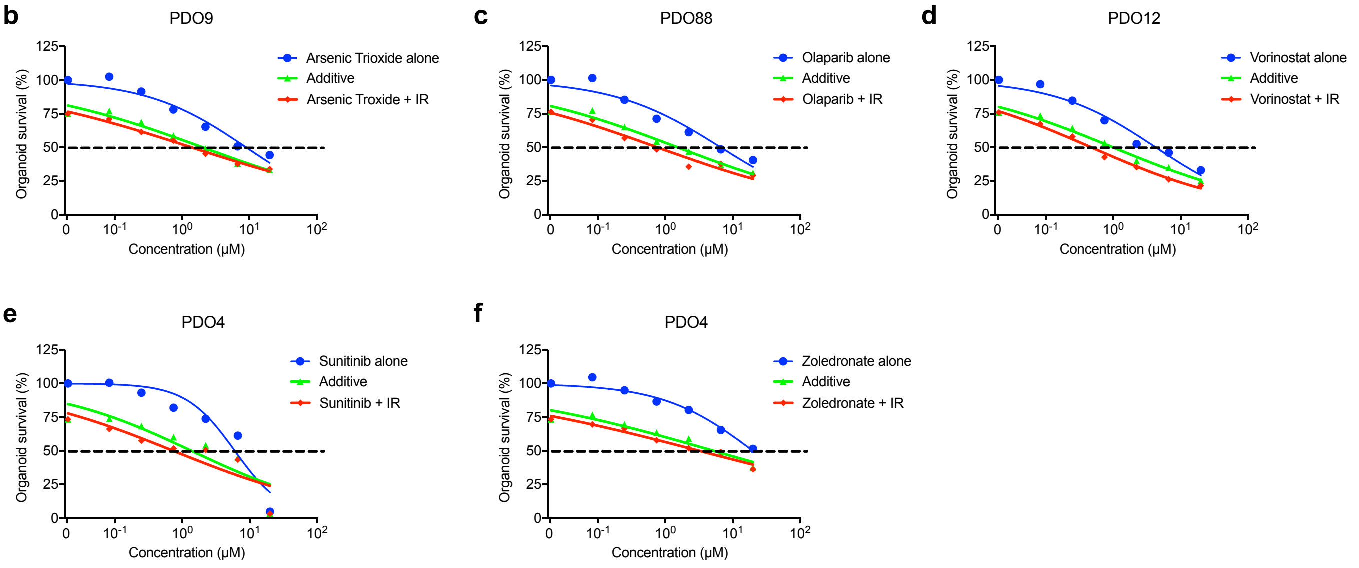

Supplementary Figure 10 Chemoradiotherapy (CRT) screening identified combinations with synergistical effect. a Therapeutic response comparison of EGFR targeting drugs between drug alone and drug combining with IR on PDOs derived from EC, MSEC, and SC subtypes. The combination between EGFRi and IR exhibited good synergy. **b-f** CRT screening identified synergistic combinations of Arsenic trioxide (2.222 μM) and IR (b); Olaparib (2.222 μM) and IR (c); Vorinostat (2.222 μM) and IR (d); Sunitinib (2.222 μM) and IR (e); Zoledronate (6.667 μM) and IR (f). The single treatment curves (b-f) are presented with blue color; the combinational treatment curves (b-f) are labelled with red color; and the additive curves (b-f) are marked with green color. The additive curves (b-f) represent the estimated combinational treatment effect being equal to the sum of their separate effects. If the combinational treatment curve (red) is lower than the additive curve (green), the synergistic effect is demonstrated. Data points (b-f) represent mean % viability relative to control.

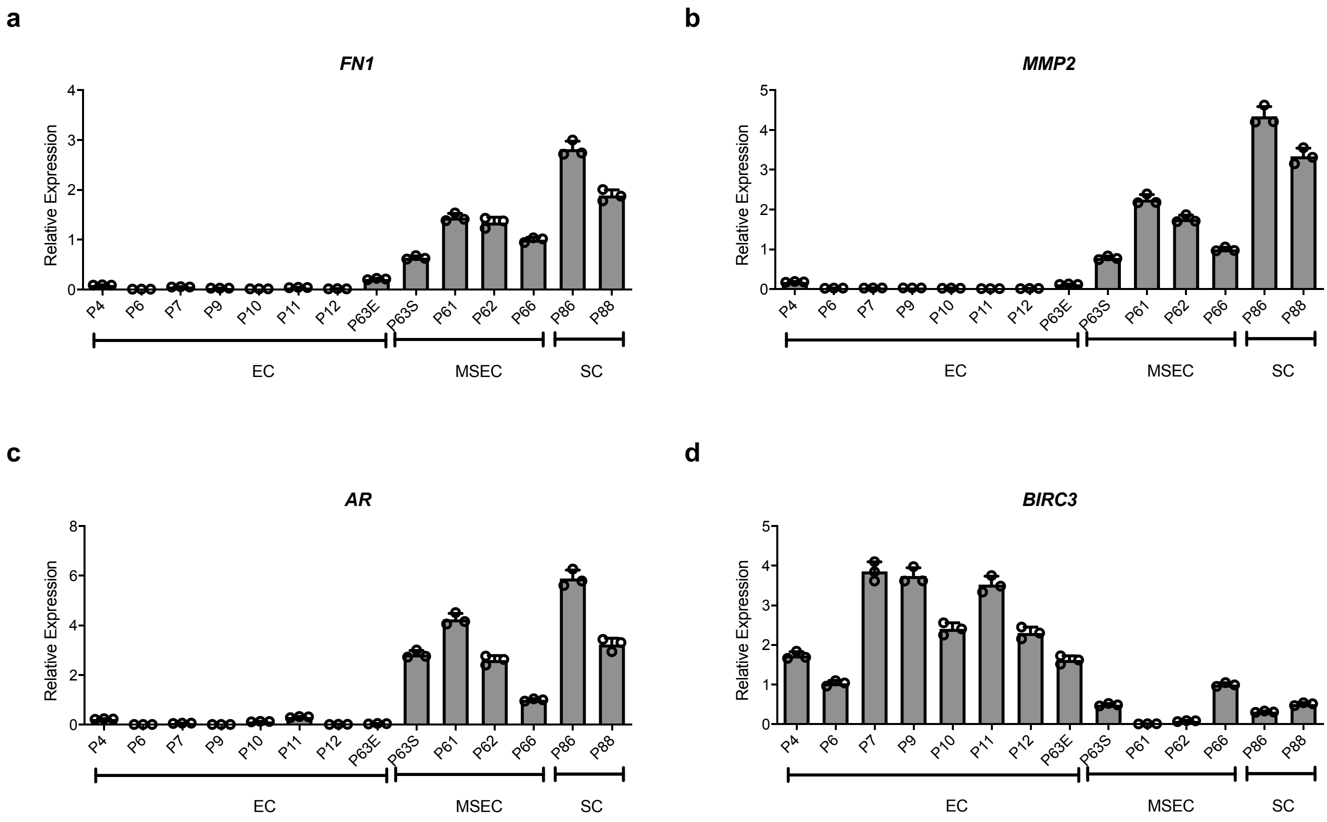

Supplementary Figure 11 qPCR validation of key genes. a *FN1*; **b** *MMP2*; **c** *AR*; **d** *BIRC3*. Data bars (a-d) represent means of relative expression fold changes relative to PDO66 ± SEM, for n = 3 technical replicates. Data points are displayed as dots. Graphs (a-d) are representatives of 2 independent experiments.

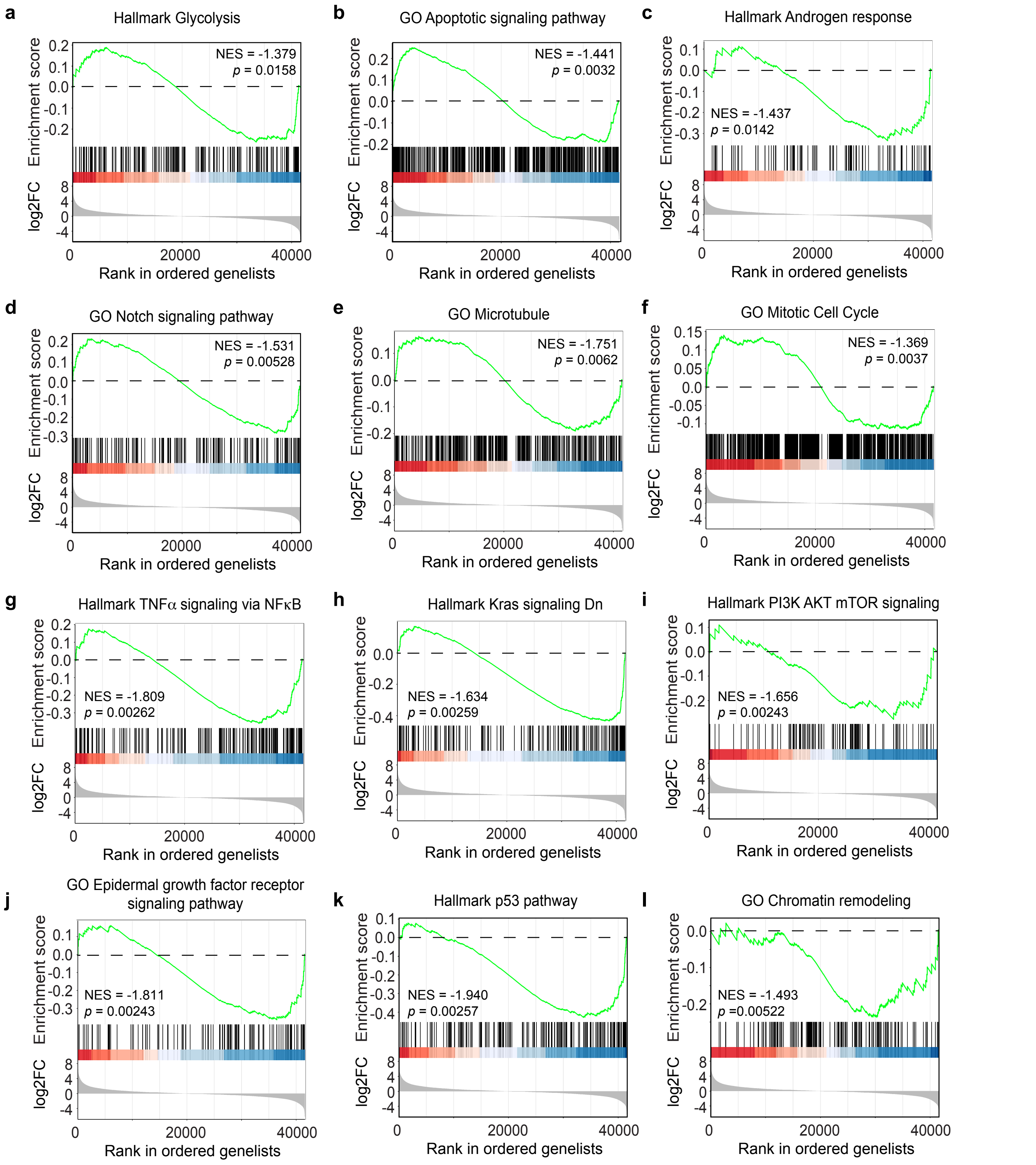

Supplementary Figure 12 Transcriptome analysis on PDOs. a-l GSEA enrichment plots for gene sets of Hallmark Glycolysis (a); GO apoptotic signaling pathway (b); Hallmark Androgen response (c); GO Notch signaling (d); GO Microtubule (e); GO Mitotic cell cycle (f); Hallmark TNFα signaling via NF-κB (g); Hallmark KRAS signaling downregulation (h); Hallmark PI3K-AKT-mTOR signaling (i); GO EGFR signaling pathway (j); Hallmark p53 pathway (k); GO Chromatin remodeling (l). Statistical significance was calculated using two-sided *t*-test. The *p* values regarding comparison between EC-type and SC-type groups for each pathway are presented.

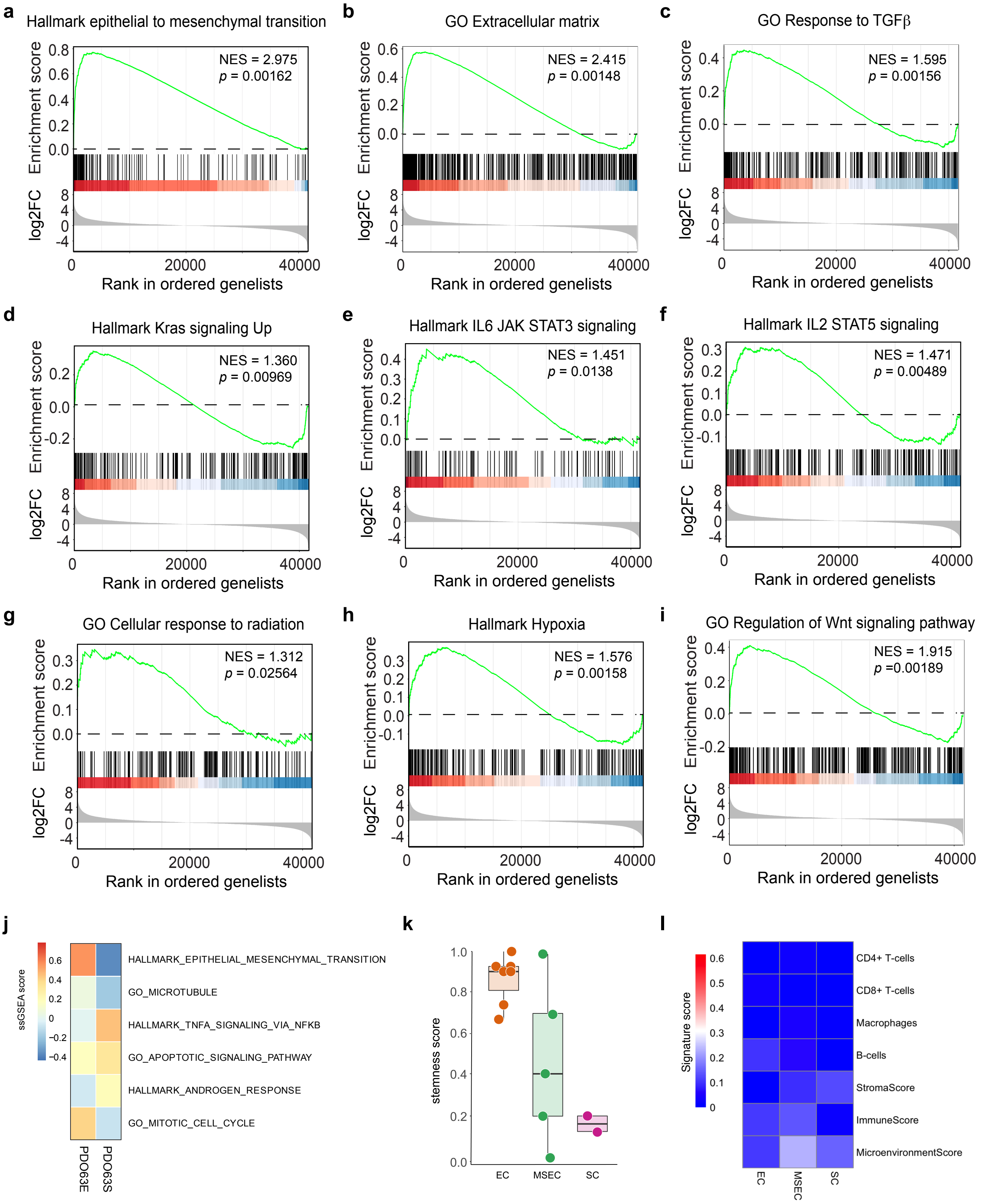

Supplementary Figure 13 Transcriptome analysis on PDOs (Continued). a-i GSEA enrichment plots for gene sets of Hallmark EMT transition (a); GO Extracellular matrix (b); GO Response to TGF-β (c); Hallmark KRAS signaling upregulation (d); Hallmark IL6-JAK-STAT3 signaling (e); Hallmark IL2-STAT5 signaling (f); GO Cellular response to radiation (g); Hallmark Hypoxia (h); GO Regulation of Wnt signaling pathway (i). Statistical significance (a-i) was calculated using two-sided *t*-test, the *p* values regarding comparison between EC-type and SC-type groups for each pathway are presented. **j** Heatmap representing ssGSEA scores of subtype-differentiated pathways on EC-type PDO63E and SC-type PDO63S organoids. **k** Comparison of stemness scores among PDOs derived from EC, MSEC and SC subtypes. Box plots represent median levels, first and third percentiles. Whiskers indicate 1.5 × inter-quartile range (IQR) extending from the hinges. Data points are displayed as dots. **l** Heatmap representing immune and other key cell type signature scores of organoids derived from EC, MSEC and SC subtypes.

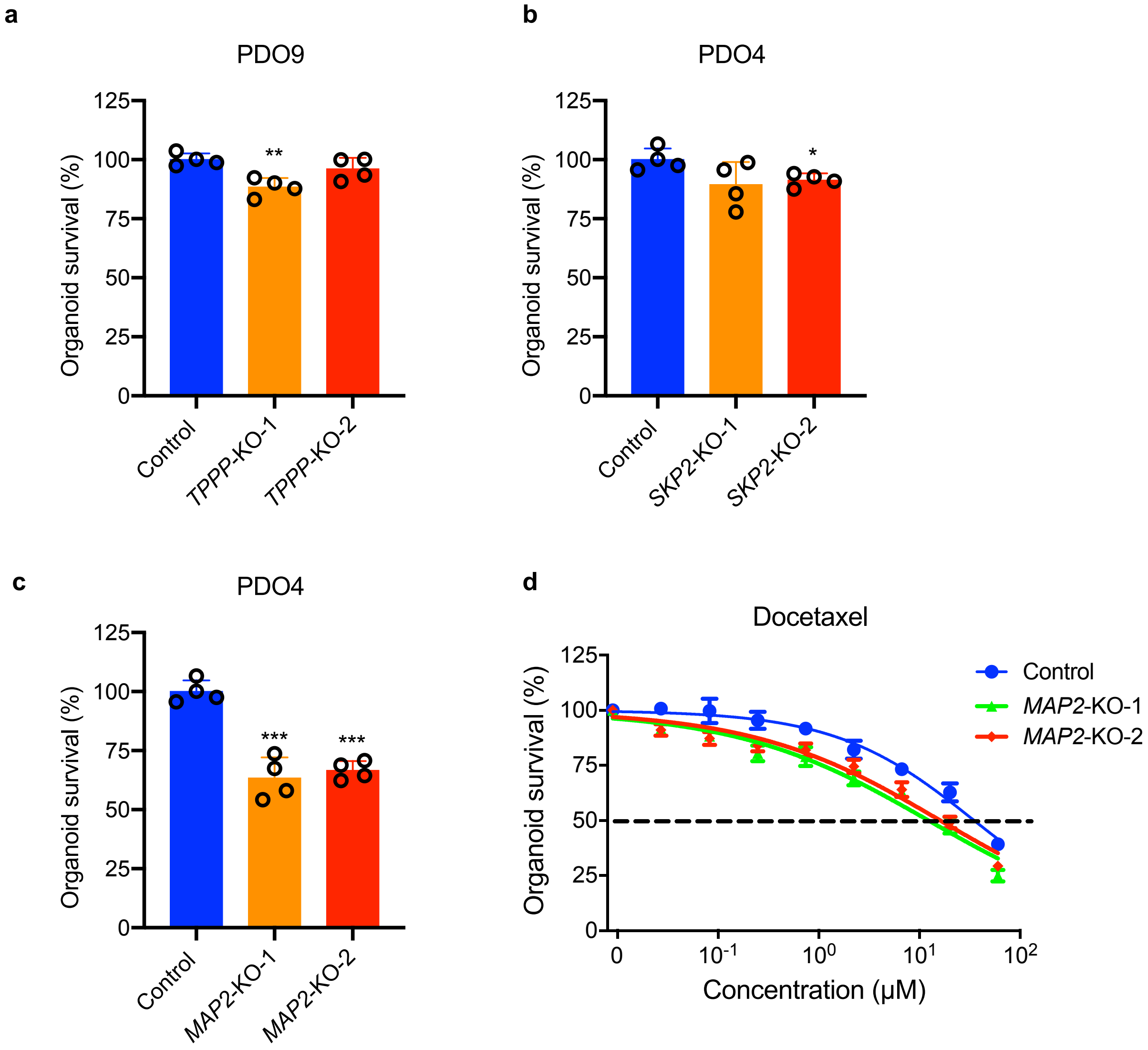

Supplementary Figure 14 Gene manipulation using CRISPR-Cas9. a Survival examination of *TPPP* knockout in PDO9. **b** Survival examination of *SKP2* knockout in PDO4. **c** Survival examination of *MAP2* knockout in PDO4. Data bars (a-c) represent mean % viability relative to control ± SEM, for n = 4 biological replicates. Data points are displayed as dots. Graphs (a-c) are representatives of 3 independent experiments. Statistical significance was calculated using two-sided *t*-test for comparing difference between nontargeting control and gene knockout groups, with * $p \leq 0.05$, ** $p \leq 0.01$, *** $p \leq 0.001$. **d** *MAP2* knockout increased sensitivity of PDO9 to docetaxel treatment. Drug response curves are presented with blue color for nontargeting control, green and red colors for gene knockout groups. Data points represent mean % viability relative to control ± SEM, for n = 3 biological replicates. Graphs are representatives of 3 independent experiments.

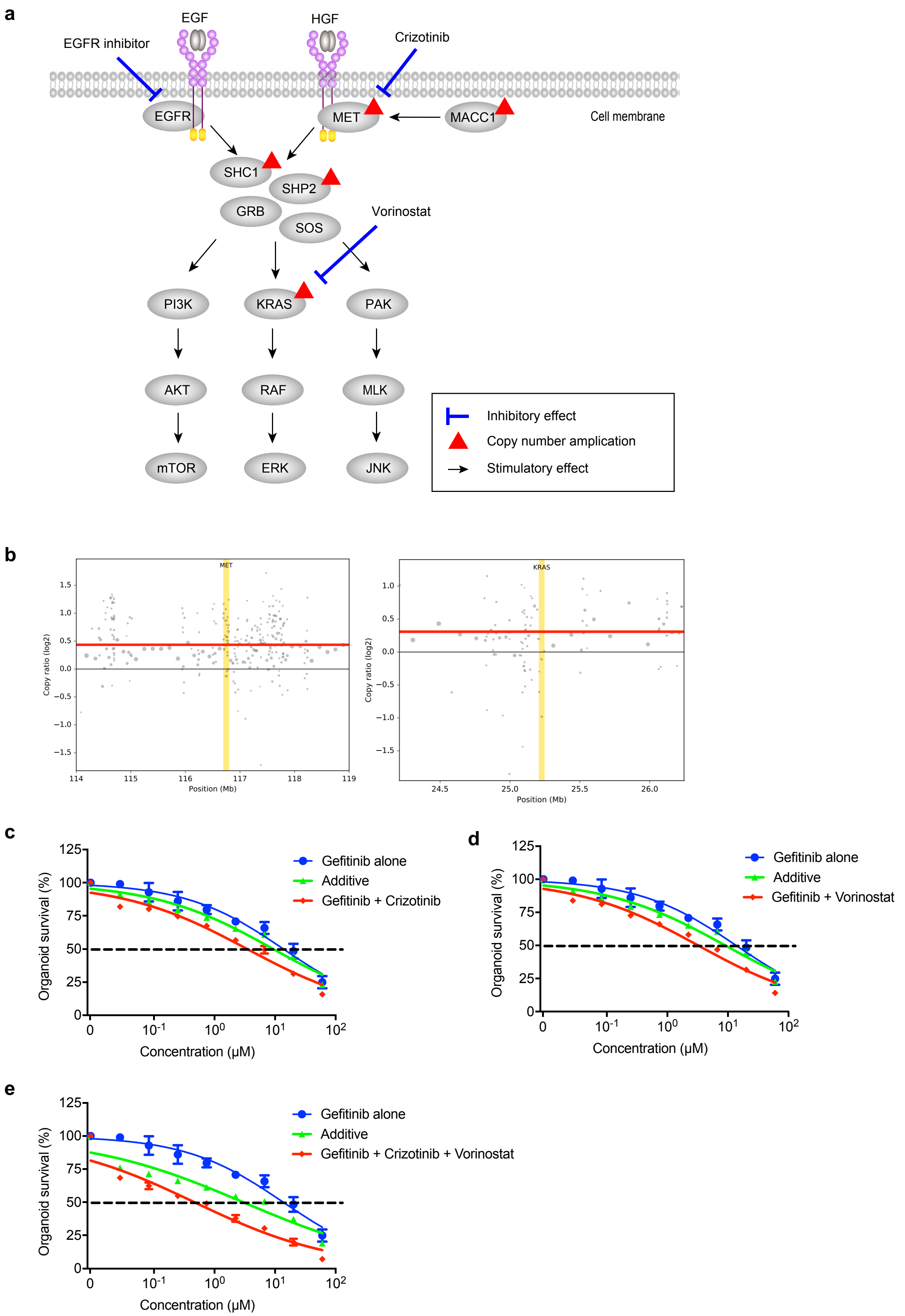
a
EGF
HGF
EGFR inhibitor
Crizotinib
Cell membrane
EGFR
MET
MACC1
SHC1
SHP2
GRB
SOS
Vorinostat
PI3K
KRAS
PAK
AKT
RAF
MLK
mTOR
ERK
JNK
Inhibitory effect
Copy number amplication
Stimulatory effect
b
MET
KRAS
Copy ratio (log2)
Position (Mb)
c
Gefitinib alone
Additive
Gefitinib + Crizotinib
Organoid survival (%)
Concentration (μM)
d
Gefitinib alone
Additive
Gefitinib + Vorinostat
Organoid survival (%)
Concentration (μM)
e
Gefitinib alone
Additive
Gefitinib + Crizotinib + Vorinostat
Organoid survival (%)
Concentration (μM)

Supplementary Figure 15 Pharmacogenomics-based precision medicine. a CNVs identified from P86 explained the mechanism of drug resistance to EGFR inhibitors in PDO86. Amplification of *MET* and *KRAS* may contribute to the drug resistance. **b** Confirmation of copy number gain events in *MET* and *KRAS* locus. **c** Combinational treatment of gefitinib and crizotinib in PDO86.The synergistic effect was observed by two drug combination. **d** Combinational treatment of gefitinib and vorinostat in PDO86. The synergistic effect was observed by two drug combination. **e** Overcoming gefitinib resistance in PDO86 by synergistically combinational treatment of gefitinib, crizotinib and vorinostat. The single treatment curves (c-e) are presented with blue color; the combinational treatment curves (c-e) are labelled with red color; and the additive curves (c-e) are marked with green color. The additive curves (c-e) represent the estimated combinational treatment effect being equal to the sum of their separate effects. If the combinational treatment curve (red) is lower than the additive curve (green), the synergistic effect is demonstrated. Data points (c-e) represent mean % viability relative to control ± SEM, for n = 3 biological replicates. Graphs (c-e) are representatives of 3 independent experiments.

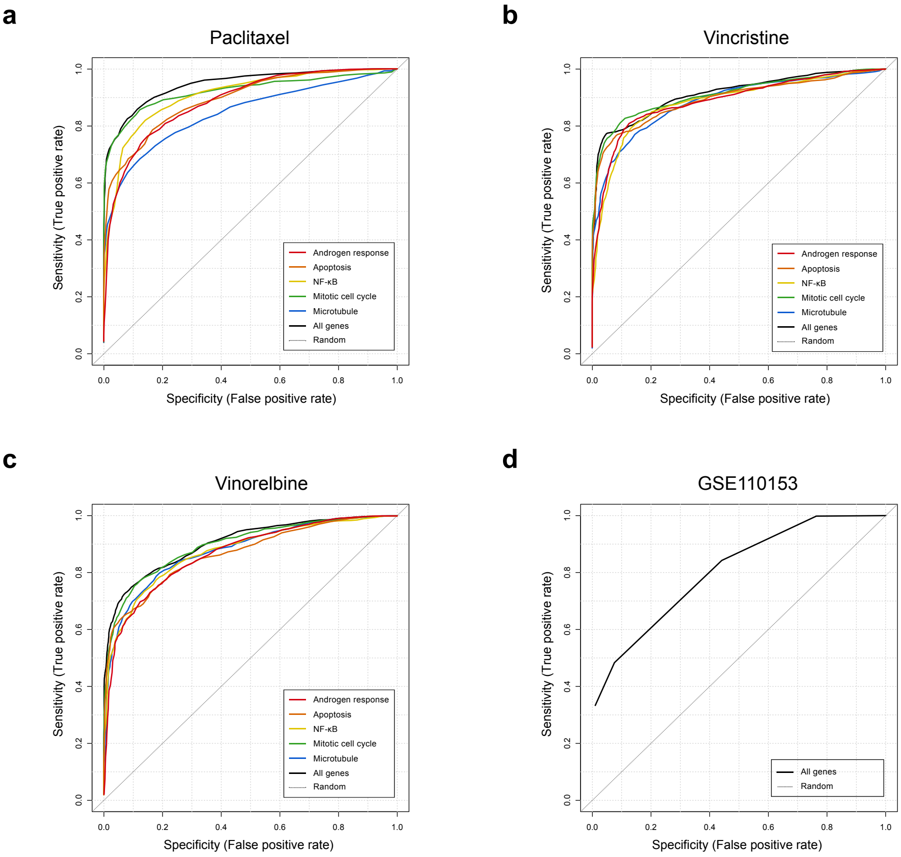

Supplementary Figure 16 Drug prediction accuracy evaluation. a Receiver operating characteristic (ROC) curves illustrating estimated paclitaxel prediction accuracy using signature gene sets of 5 key correlated pathways, including all genes (black, area under curve (AUC) =0.949), GO apoptotic signaling pathway (orange, AUC=0.899), GO Microtubule (blue, AUC=0.848), GO Mitotic cell cycle (green, AUC=0.927), Hallmark Androgen response (red, AUC=0.895), Hallmark TNFα signaling via NF-κB (yellow, AUC=0.913). **b** ROC curves illustrating estimated vincristine prediction accuracy using signature gene sets of 5 key correlated pathways, including all genes (black, AUC=0.915), GO apoptotic signaling pathway (orange, AUC=0.895), GO Microtubule (blue, AUC=0.886), GO Mitotic cell cycle (green, AUC=0.914), Hallmark Androgen response (red, AUC=0.893), Hallmark TNFα signaling via NF-κB (yellow, AUC=0.885). **c** ROC curves illustrating estimated vinorelbine prediction accuracy using signature gene sets of 5 key correlated pathways, including all genes (black, AUC=0.906), GO apoptotic signaling pathway (orange, AUC=0.87), GO Microtubule (blue, AUC=0.88), GO Mitotic cell cycle (green, AUC=0.903), Hallmark Androgen response (red, AUC=0.869), Hallmark TNFα signaling via NF-κB (yellow, AUC=0.876). **d** ROC curve illustrating estimated docetaxel prediction accuracy using all signature genes from 5 key pathways (black, AUC=0.872) on GSE110153 dataset of human breast cancer patient derived xenografts.

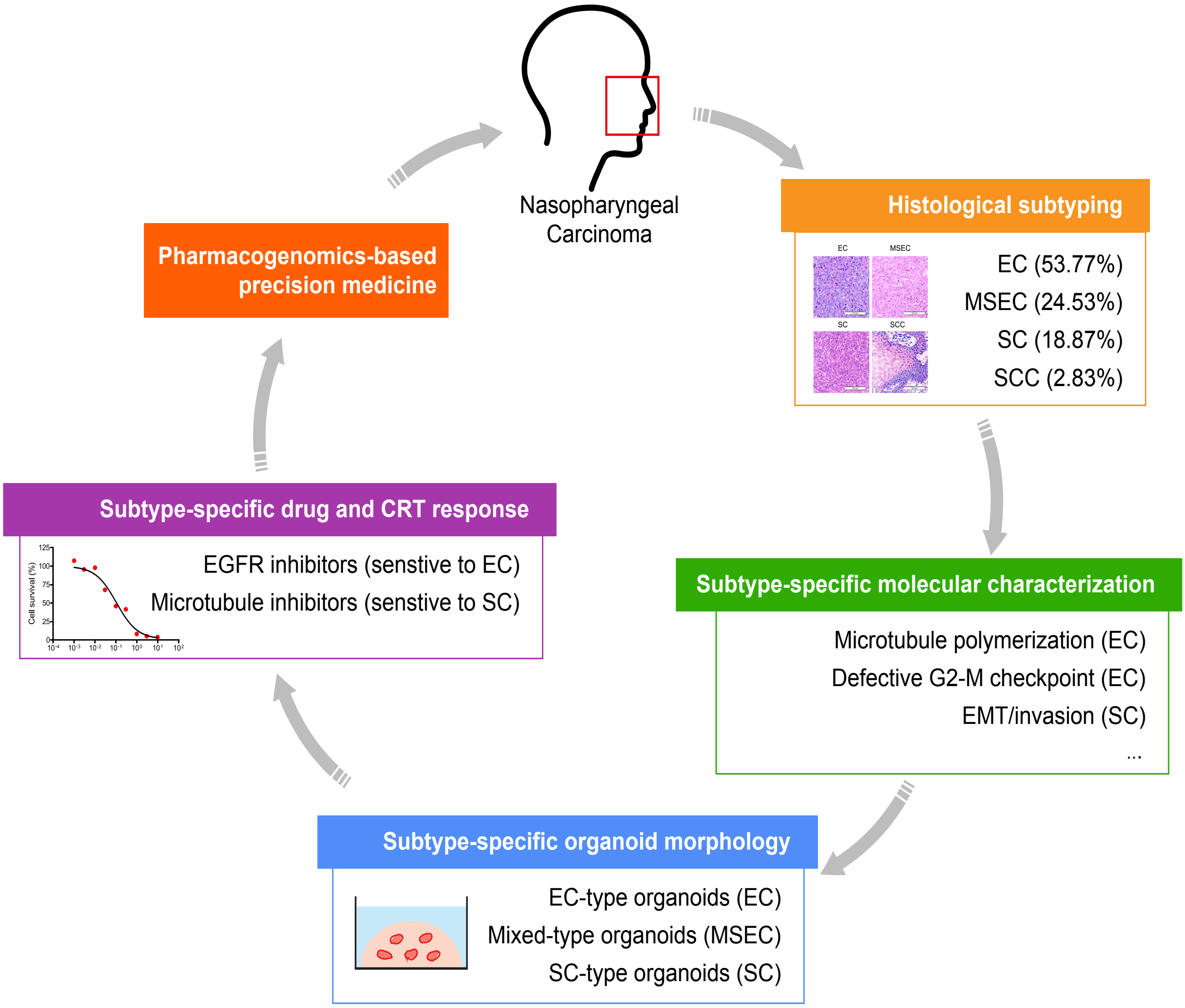

Supplementary Figure 17 Graphical summary. This study offers subtype-specific molecular characterizations for EC, MSEC and SC subtypes, develops patient-derived organoid models for NPC. We also identify subtype-specific chemotherapy and chemoradiotherapy responses for NPC, which are well correlated with corresponding subtype-specific molecular features. Finally, a pipeline for integrative pharmacogenomics-based precision medicine approach is proposed to facilitate precision oncology.

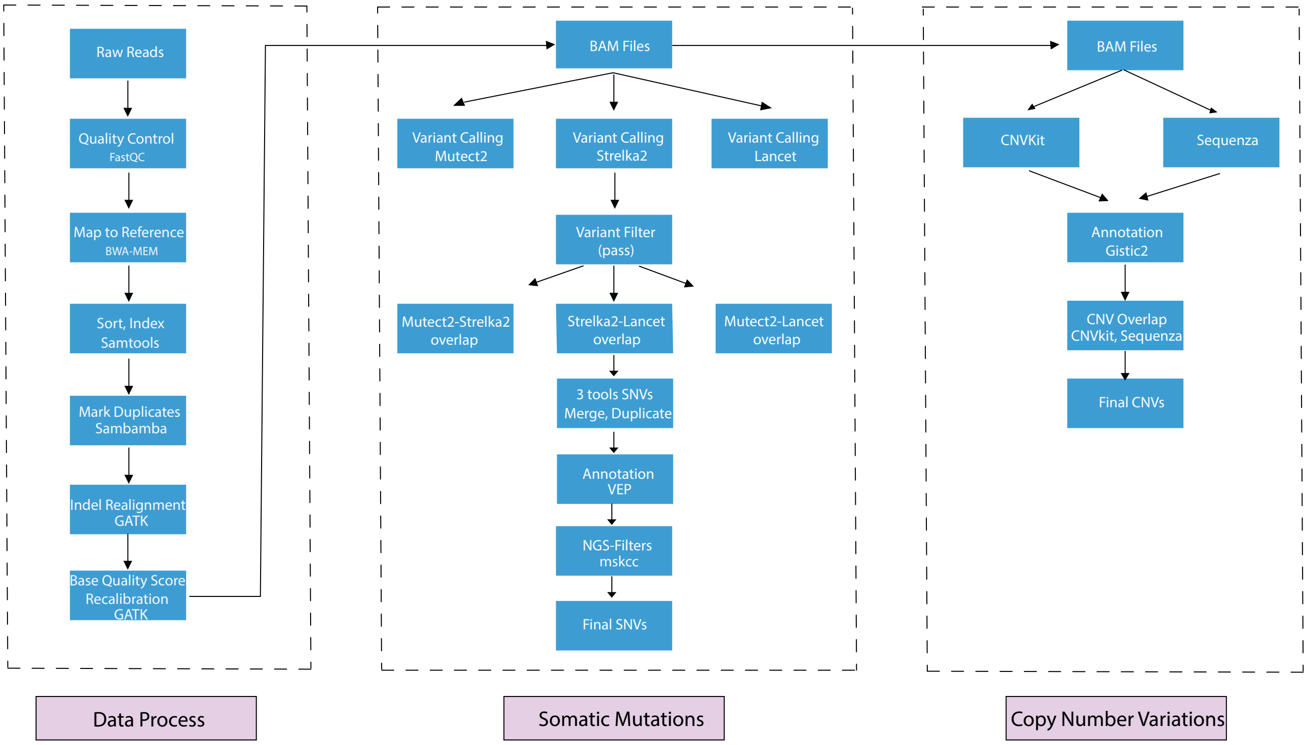

Supplementary Figure 18 Workflow of genomic data processing. Sequence reads were aligned to human genome build 38 (Hg38) using Burrows-Wheeler Alignment tool (BWA-version 0.7.12) (20080505) with maximal exact matches (v0.7.17) [1] followed by reducing duplicates with Sambamba (v0.6.8) [2], realignment of indels and base recalibration with Genome Analysis ToolKit (v4.1.0.0) [3] according to the best practice guidelines. Somatic SNVs and INDELs were called by three independent programs with default parameters, including MuTect2 (v4.1.0.0), Strelka2 (v2.9.10) [4], and LANCET (v1.1.0) [5] (Supplementary Fig. 2A). Paired blood control or adjacent control samples were applied as the reference to call somatic mutations of each tumor independently. The results of each caller were stored in VCF format and further filtered for PASS variants. The vcf2maf tools (https://github.com/mskcc/vcf2maf) was used to convert VCF into MAF with performing annotation by ENSEMBL Variant Effect Predictor (v100.0) [6] according to reference release 93. Only mutations detected by at least 2 callers were kept as true positive ones and assigned for further analysis. Somatic SNVs and INDELs were further filtered by ngs-filters (https://github.com/mskcc/ngs-filters) to determine high-confident variants. Copy number analysis was performed in both Sequenza (v3.0.0) [7], and CNVkit (v0.9.0) [8] with default parameter. Further chromosome arm-level and gene-level variation were detected by GISTIC 2.0 [9]. Consensus CNVs detected by both CNVkit and Sequenza were considered as tune positive ones.

Supplementary Table 1 Clinicopathological characteristics of NPC cohort (n=106)

Characteristics	No. of Patients	Percentage (%)
**Gender**		
Male	72	67.92
Female	34	32.08
**Age of Diagnosis**		
<50years	58	54.72
≥50 years	48	45.28
**Ethnicity**		
Chinese	106	100.00
**Tumor stage**		
I	13	13.54
II	25	26.04
III	43	44.79
IV	15	15.63
No information	10	-
**Histological Subtype**		
EC	57	53.77
MSEC	26	24.53
SC	20	18.87
SCC	3	2.83
**WHO Subtype**		
NKUC	78	73.58
NKDC	25	23.58
KSCC	3	2.83
**Node Classification**		
0	13	13.54
1	27	28.13
2	41	42.71
3	14	14.58
4	1	1.04
No information	10	-
**Metastasis Status**		
Yes	18	18.75
No	78	81.25
No information	10	-
**Smoking Habit**		
Yes	18	21.18
Early Smoker	6	7.06
No	61	71.76
No information	21	-
**EBV status**		
Strongly positive	36	90.00
Low positive	4	10.00
Not determined	66	-

Supplementary Table 2 Comparison of driver SNV frequencies in NPC

Gene (SNV)	Our study (n=88)	Li et al, 2017 (n=111) [10]	Lin et al, 2014 (n=113) [11]	Zheng et al, 2016 (n=124) [12]	Zhang et al, 2017 (n=51) [13]
TP53	8.0%	9.0%	10.6%	7.3%	5.9%
CYLD	10.2%	9.9%	-	3.2%	5.9%
KMT2C	5.7%	5.4%	-	3.2%	2.0%
FBXW7	4.5%	3.6%	-	-	3.9%
NOTCH2	6.8%	2.7%	0.9%	-	-
NFKBIA	4.5%	6.3%	-	4.8%	-
TRAF3	3.4%	8.1%	-	-	5.9%
PTEN	2.3%	3.6%	-	2.4%	-
MTOR	2.3%	1.8%	1.8%	-	-
PIK3C2B	2.3%	-	0.9%	-	-
PIK3CG	2.3%	-	1.8%	-	-
KMT2B	3.4%	1.8%		2.4%	-
ARID1A	2.3%	-	4.4%	1.6%	-
NF1	4.5%	1.8%	-	-	-
ATM	3.4%	-	-	1.6%	-
BAP1	2.3%	-	3.5%	3.2%	-
SYNE1	2.3%	-	8.9%	-	2.0%
FAT2	3.4%	-	2.7%	-	-

"-" refers to unmentioned.

Supplementary Table 3 Comparison of recurrent chromosomal loss frequencies in NPC

		Our study (n=99)	Li et al, 2017 (n=111) [10]	Chen et al, 1999 (n=51) [14]	Hui et al, 1999, (n=20) [15]	Fang et al, 2001, (n=47) [16]	Chien et al, 2001 (n=30) [17]	Yan et al, 2001 (n=17) [18]	Rodriguez et al, 2005 (n=10) [19]	Yan et al, 2005 (n=32) [20]
Loss	Gene	Copy number loss frequencies (WES)		Chromosomal alteration frequencies (comparative genomic hybridization)						
3p	MST1R (3p21.31)	55.6%	-	53%	75%	43%	20%	53%	40%	34%
	BAP1 (3p21.1)	55.6%	-							
9p	CDKN2A (9p21.3)	32.3%	42.3%	41%	25%	-	-	-	20%	25%
	CDKN2B (9p21.3)	31.3%	42.3%							
11q	BIRC2 (11q22.2)	30.3%	-	29%	45%	36%	23%	47%	40%	31%
	BIRC3 (11q22.2)	30.3%	-							
14q	NFKBIA (14q13.2)	43.4%	21.6%	35%	65%	21%	13%	47%	40%	47%
	TRAF3 (14q32.32)	57.6%	22.5%							
16q	CYLD (16q12.1)	16.2%	28.3%	-	50%	55%	16%	29%	30%	34%
	NLRC5 (16q13)	16.2%	21.6%							
17p	TP53 (17p13.1)	20.2%	16.2%	-	-	-	-	-	-	31%

"-" refers to unmentioned.

Supplementary Table 4 Comparison of recurrent chromosomal gain frequencies in NPC

		Our study (n=99)	Li et al, 2017 (n=111) [10]	Chen et al, 1999 (n=51) [14]	Hui et al, 1999, (n=20) [15]	Fang et al, 2001, (n=47) [16]	Chien et al, 2001 (n=30) [17]	Yan et al, 2001 (n=17) [18]	Rodriguez et al, 2005 (n=10) [19]	Yan et al, 2005 (n=32) [20]
Gain	Gene	Copy number gain frequencies (WES)		Chromosomal gain frequencies (comparative genomic hybridization)						
3q	PIK3CA (3q26.32)	8.1%	10.8%	-	30%	34%	20%	29%	30%	34%
7q	MET (7q31)	11.1%	-	-	20%	-	13%	-	10%	-
	BRAF (7q34)	12.1%	-							
	KMT2C (7q36.1)	12.1%	11.7%							
8q	RIPK2 (8q21.3)	12.1%	-	-	30%	-	27%	29%	10%	47%
	NIBP (8q24.3)	17.2%	-							
11q	CCND1 (11q13.3)	15.2%	44.1%	41%	-	-	-	35%	20%	-
12p	KRAS (12p12.1)	31.3%	24.3%	59%	60%	-	36%	-	40%	41%
	TNFRSF1A (12p13.31)	28.3%	-							
	LTBR (12p13.31)	27.3%	-							
12q	ERBB3 (12q13.2)	23.2%	-	35%	60%	51%	33%	41%	10%	53%
	KMT2D (12q13.12)	21.2%	9.0%							

"-" refers to unmentioned.

Supplementary Table 5 Overview of organoid derivation

	Organoid ID	Tumor ID	Organoid morphology	Tumor subtype	Tested maximum passage	Doubling time (hours)	Drug screening	DNA-seq	RNA-seq
1	PDO3	P3	EC-type	EC	9	77.9	Yes	No	No
2	PDO4	P4	EC-type	EC	25	42.3	Yes	No	Yes
3	PDO5	P5	EC-type	EC	7	67.5	Yes	No	No
4	PDO6	P6	EC-type	EC	8	103	Yes	No	Yes
5	PDO7	P7	EC-type	EC	12	83	Yes	No	Yes
6	PDO8	P8	EC-type	EC	2	112	Yes	No	No
7	PDO9	P9	EC-type	EC	22	44.2	Yes	Yes	Yes
8	PDO10	P10	EC-type	EC	10	65.3	Yes	No	Yes
9	PDO11	P11	EC-type	EC	22	40.3	Yes	Yes	Yes
10	PDO12	P12	EC-type	EC	21	67.1	Yes	Yes	Yes
11	PDO13	P13	EC-type	EC	18	88.9	Yes	No	No
12	PDO14	P14	EC-type	EC	15	81	Yes	No	No
13	PDO15	P15	EC-type	EC	15	88.1	Yes	No	No
14	PDO16	P16	EC-type	EC	5	48.2	Yes	No	No
15	PDO17	P17	EC-type	EC	5	59.7	Yes	No	No
16	PDO18	P18	EC-type	EC	5	69.1	Yes	No	No
17	PDO50	P50	EC-type	EC	5	-	Yes	Yes	No
18	PDO51	P51	EC-type	EC	5	-	Yes	No	No
19	PDO53	P53	EC-type	EC	5	-	Yes	Yes	No
20	PDO54	P54	EC-type	EC	5	-	Yes	No	No
21	PDO55	P55	EC-type	EC	5	-	Yes	Yes	No
22	PDO56	P56	EC-type	EC	5	-	Yes	No	No
23	PDO57	P57	EC-type	EC	5	-	Yes	Yes	No
24	PDO61	P61	SC-type	MSEC	18	108	Yes	No	Yes
25	PDO62	P62	SC-type	MSEC	27	48.3	Yes	No	Yes
26	PDO63E	P63	EC-type	MSEC	22	66.7	Yes	No	Yes
27	PDO63S	P63	SC-type	MSEC	30	59.4	Yes	No	Yes
28	PDO64	P64	SC-type	MSEC	18	62.3	Yes	No	No
29	PDO65	P65	SC-type	MSEC	13	82.2	Yes	No	No
30	PDO66E	P66	EC-type	MSEC	5	71.8	Yes	Yes	No
31	PDO66S	P66	SC-type	MSEC	12	46.2	Yes	Yes	Yes
32	PDO68	P68	SC-type	MSEC	5	70.9	Yes	No	No
33	PDO81	P81	SC-type	MSEC	5	-	Yes	No	No
34	PDO82	P82	SC-type	MSEC	5	-	Yes	No	No
35	PDO83	P83	SC-type	MSEC	5	-	Yes	Yes	No
36	PDO86	P86	SC-type	SC	28	94.2	Yes	No	Yes
37	PDO87	P87	SC-type	SC	24	79.1	Yes	Yes	No
38	PDO88	P88	SC-type	SC	23	71.3	Yes	Yes	Yes
39	PDO89	P89	SC-type	SC	11	53.8	Yes	Yes	No
40	PDO90	P90	SC-type	SC	5	80.3	Yes	No	No
41	PDO102	P102	SC-type	SC	5	-	Yes	Yes	No
42	PDO103	P103	SC-type	SC	5	-	Yes	Yes	No

"-" refers to unevaluated.

Supplementary Table 6 Primers used for qPCR validation

Gene name	Forward primer	Reverse primer
FN1	CGGTGGCTGTCAGTCAAAG	AAACCTCGGCTTCCTCCATAA
MMP2	CCCACTGCGGTTTTCTCGAAT	CAAAGGGGTATCCATCGCCAT
AR	GACGACCAGATGGCTGTCATT	GGGCGAAGTAGAGCATCCT
BIRC3	AAGCTACCTCTCAGCCTACTTT	CCACTGTTTTCTGTACCCGGA

Reference

1. Li, H. & Durbin, R. Fast and accurate long-read alignment with Burrows-Wheeler transform. *Bioinformatics* **26**, 589-595 (2010).
2. Tarasov, A., Vilella, A.J., Cuppen, E., Nijman, I.J. & Prins, P. Sambamba: fast processing of NGS alignment formats. *Bioinformatics* **31**, 2032-2034 (2015).
3. McKenna, A. *et al.* The Genome Analysis Toolkit: a MapReduce framework for analyzing next-generation DNA sequencing data. *Genome Res* **20**, 1297-1303 (2010).
4. Kim, S. *et al.* Strelka2: fast and accurate calling of germline and somatic variants. *Nat Methods* **15**, 591-594 (2018).
5. Narzisi, G. *et al.* Genome-wide somatic variant calling using localized colored de Bruijn graphs. *Commun Biol* **1** (2018).
6. McLaren, W. *et al.* The Ensembl Variant Effect Predictor. *Genome Biol* **17**, 122 (2016).
7. Favero, F. *et al.* Sequenza: allele-specific copy number and mutation profiles from tumor sequencing data. *Annals of oncology : official journal of the European Society for Medical Oncology* **26**, 64-70 (2015).
8. Talevich, E., Shain, A.H., Botton, T. & Bastian, B.C. CNVkit: Genome-Wide Copy Number Detection and Visualization from Targeted DNA Sequencing. *PLoS Comput Biol* **12**, e1004873 (2016).
9. Mermel, C.H. *et al.* GISTIC2.0 facilitates sensitive and confident localization of the targets of focal somatic copy-number alteration in human cancers. *Genome Biol* **12**, R41 (2011).
10. Li, Y.Y. *et al.* Exome and genome sequencing of nasopharynx cancer identifies NF-kappaB pathway activating mutations. *Nat Commun* **8**, 14121 (2017).
11. Lin, D.C. *et al.* The genomic landscape of nasopharyngeal carcinoma. *Nat Genet* **46**, 866-871 (2014).
12. Zheng, H. *et al.* Whole-exome sequencing identifies multiple loss-of-function mutations of NF-κB pathway regulators in nasopharyngeal carcinoma. *Proc Natl Acad Sci U S A* **113**, 11283-11288 (2016).
13. Zhang, L. *et al.* Genomic Analysis of Nasopharyngeal Carcinoma Reveals TME-Based Subtypes. *Mol Cancer Res* **15**, 1722-1732 (2017).
14. Chen, Y.J. *et al.* Chromosomal aberrations in nasopharyngeal carcinoma analyzed by comparative genomic hybridization. *Genes, chromosomes & cancer* **25**, 169-175 (1999).
15. Hui, A.B. *et al.* Detection of recurrent chromosomal gains and losses in primary nasopharyngeal carcinoma by comparative genomic hybridisation. *International journal of cancer* **82**, 498-503 (1999).
16. Fang, Y. *et al.* Analysis of genetic alterations in primary nasopharyngeal carcinoma by comparative genomic hybridization. *Genes, chromosomes & cancer* **30**, 254-260 (2001).
17. Chien, G., Yuen, P.W., Kwong, D. & Kwong, Y.L. Comparative genomic hybridization analysis of nasopharygeal carcinoma: consistent patterns of genetic aberrations and clinicopathological correlations. *Cancer genetics and cytogenetics* **126**, 63-67 (2001).
18. Yan, J., Fang, Y., Liang, Q., Huang, Y. & Zeng, Y. Novel chromosomal alterations detected in primary nasopharyngeal carcinoma by comparative genomic hybridization. *Chinese medical journal* **114**, 418-421 (2001).
19. Rodriguez, S. *et al.* Conventional and array-based comparative genomic hybridization analysis of nasopharyngeal carcinomas from the Mediterranean area. *Cancer genetics and cytogenetics*

157, 140-147 (2005).

20. Yan, W. *et al.* Chromosomal abnormalities associated with neck nodal metastasis in nasopharyngeal carcinoma. *Tumour biology : the journal of the International Society for Oncodevelopmental Biology and Medicine* **26**, 306-312 (2005).
