# Supplementary material for: Molecular landscape and subtype-specific therapeutic response of nasopharyngeal carcinoma revealed by integrative pharmacogenomics
Source: Nat Commun. 2021 May 24;12:3046. doi: 10.1038/s41467-021-23379-3 (PMC8144567; doi:10.1038/s41467-021-23379-3)
Supplement: Supplementary file 18 — Reporting summary [file 41467_2021_23379_MOESM18_ESM.pdf]

nature research

Corresponding author(s): Chu-Xia Deng
Last updated by author(s): Mar 17, 2021

# Reporting Summary

Nature Research wishes to improve the reproducibility of the work that we publish. This form provides structure for consistency and transparency in reporting. For further information on Nature Research policies, see Authors & Referees and the Editorial Policy Checklist .

## Statistics

For all statistical analyses, confirm that the following items are present in the figure legend, table legend, main text, or Methods section.

| n/a | Confirmed | |
|---|---|---|
| ☐ | ☒ | The exact sample size (*n*) for each experimental group/condition, given as a discrete number and unit of measurement |
| ☐ | ☒ | A statement on whether measurements were taken from distinct samples or whether the same sample was measured repeatedly |
| ☐ | ☒ | The statistical test(s) used AND whether they are one- or two-sided<br>*Only common tests should be described solely by name; describe more complex techniques in the Methods section.* |
| ☒ | ☐ | A description of all covariates tested |
| ☒ | ☐ | A description of any assumptions or corrections, such as tests of normality and adjustment for multiple comparisons |
| ☐ | ☒ | A full description of the statistical parameters including central tendency (e.g. means) or other basic estimates (e.g. regression coefficient) AND variation (e.g. standard deviation) or associated estimates of uncertainty (e.g. confidence intervals) |
| ☐ | ☒ | For null hypothesis testing, the test statistic (e.g. *F*, *t*, *r*) with confidence intervals, effect sizes, degrees of freedom and *P* value noted<br>*Give P values as exact values whenever suitable.* |
| ☒ | ☐ | For Bayesian analysis, information on the choice of priors and Markov chain Monte Carlo settings |
| ☒ | ☐ | For hierarchical and complex designs, identification of the appropriate level for tests and full reporting of outcomes |
| ☒ | ☐ | Estimates of effect sizes (e.g. Cohen's *d*, Pearson's *r*), indicating how they were calculated |

*Our web collection on statistics for biologists contains articles on many of the points above.*

## Software and code

Policy information about availability of computer code

| | |
|---|---|
| Data collection | OLYMPUS cellSens Dimension software (v1.18) was used for bright field and fluorescence images acquisition; BioRad Image Lab software (v6.0.1) was used for Western blot images acquisition; |
| Data analysis | GraphPad Prism (v 7.0) was used for generating drug response curves, calculating IC50 values and statistical significance of the drug treatment data; Burrows-Wheeler Alignment tool (v0.7.12) was used to do sequence alignment; Sambamba (v0.6.8) was used to reduce the duplicate reads; Genome Analysis ToolKit (v4.1.0.0) was sued to do realignment of indels and base recalibration; MuTect2 (v4.1.0.0), Strelka2 (v2.9.10) 81, and LANCET (v1.1.0) were used to call somatic SNVs and INDELs; The vcf2maf tools (https://github.com/mskcc/vcf2maf) was used to convert VCF into MAF with performing annotation by ENSEMBL Variant Effect Predictor (v100.0) according to reference release 93. The ngs-filters (https://github.com/mskcc/ngs-filters) was used to filtered high-confident somatic variants. Mutation signature analysis was performed by MutationalPatterns (v2.0.0) R package and further compared with COSMIC mutational signatures version 2 using deconstructSigs (v1.8.0) R package applying 'exome2genome' normalized method. Sequenza (v3.0.0) and CNVkit (v0.9.0) were used to do copy number analysis. GISTIC (v2.0 87) was used to detect chromosome arm-level and gene-level variations. Sequenza (v3.0.0) and Absolute (v1.0.6) were used to assess tumor purity. MultiQC (v1.5) was used to do quality check for RNA sequencing data; HISAT2 (v2.1.0) was used to do RNA reads alignment to human reference genome GRCh38; featureCounts program in Subread package (v1.6.4) was used to count RNA reads; DESeq2 (v1.26.0) and limma (v3.42.2) were used to identify differentially expressed genes between EC- and SC-type PDOs; The multiple hypothesis testing was performed using Benjamin-Hochberg correction implemented in the DESeq2 (v1.26.0) and limma (v3.42.2) package; R package clusterProfiler (v3.14.3) was used to do pathway enrichment analysis and gene set enrichment analysis; MetaboAnalyst (v4.0) online tool (https://www.metaboanalyst.ca) was used to do ROC analysis to evaluate drug prediction accuracy; R studio (v3.5.1) was used for data analysis and R code used in this study is available at https://github.com/xueyinglyu/DengLab-NPC-genomic-analysis. |

For manuscripts utilizing custom algorithms or software that are central to the research but not yet described in published literature, software must be made available to editors/reviewers. We strongly encourage code deposition in a community repository (e.g. GitHub). See the Nature Research guidelines for submitting code & software for further information.

## Data

Policy information about availability of data

All manuscripts must include a data availability statement. This statement should provide the following information, where applicable:

- Accession codes, unique identifiers, or web links for publicly available datasets
- A list of figures that have associated raw data
- A description of any restrictions on data availability

The WES raw data that support the finding of this study is available at Sequence Read Archive (SRA) database under the accession number PRJNA716262 (https://www.ncbi.nlm.nih.gov/bioproject/PRJNA716262/) and the National Omics Data Encyclopedia (NODE) under accession number OEP001733 (https://www.biosino.org/node/project/detail/OEP001733/); The RNA sequencing raw data is available at Sequence Read Archive (SRA) database under the accession number PRJNA682500 (https://www.ncbi.nlm.nih.gov/bioproject/PRJNA682500/); Public data analyzed in this paper were download from Genomics of Drug Sensitivity in Cancer (GDSC) database (https://www.cancerrxgene.org/) and Gene Expression Omnibus (GEO) database (https://www.ncbi.nlm.nih.gov/geo/). Drug response (docetaxel, paclitaxel, vincristine and vinorelbine) and transcriptional data of cancer cell lines are available from GDSC (https://www.cancerrxgene.org/downloads/bulk_download). Drug sensitivity (docetaxel) and transcriptional data of PDX were download from GEO under accession number GSE110153 (https://www.ncbi.nlm.nih.gov/geo/query/acc.cgi?acc=GSE110153). Treatment response (paclitaxel, docetaxel) and transcriptional data of cancer patients were download from GEO under accession number GSE22513 (https://www.ncbi.nlm.nih.gov/geo/query/acc.cgi?acc=GSE22513) and GSE6434 (https://www.ncbi.nlm.nih.gov/geo/query/acc.cgi?acc=GSE6434). All the other relevant data supporting the key findings of this study can be found within the supplementary files.

# Field-specific reporting

Please select the one below that is the best fit for your research. If you are not sure, read the appropriate sections before making your selection.

☒ Life sciences ☐ Behavioural & social sciences ☐ Ecological, evolutionary & environmental sciences

For a reference copy of the document with all sections, see nature.com/documents/nr-reporting-summary-flat.pdf

# Life sciences study design

All studies must disclose on these points even when the disclosure is negative.

| | |
|---|---|
| Sample size | The total tumor sample size was determined by quantity of human participate involved in. In present study, we collected samples from 106 NPC patients to conduct WES sequence and the sample size is recognized by the filed to support a good-quality genomics characterization. The sample size for further WES analysis was determined by if samples were with enough sequencing coverages (>10X) and were with paired normal control or not. Only paired samples were used for somatic SNV identification. Only samples with sequencing coverages over 10X were used for CNV and SNV calling. The organoid sample size was determined by the availability of fresh tumor samples and the success rate of organoid culture establishment. Generally, we assigned most of the available fresh tumors to do organoid culture, and totally established 42 patient-derived organoid (PDO) lines. All established organoid lines were later assigned for drug library screening. For genomics comparison between PDOs and parental tumors, we used 15 pairs to provide representative demonstration. For NPC subtype characterization, we used all 106 tumor slides to perform H&E staining. For immunohistochemistry staining experiments, we used at least 6 samples for each subtype/group, which is sufficient to demonstrate the representative results. Because SCC subtype only contained 3 samples, we used all of them. For Western blot experiment regarding gefitinib and docetaxel treatment, we used 13 PDO lines consist of at least 6 samples for sensitive and 6 samples for resistant groups, which is sufficient to demonstrate the representative results. For RNA sequencing analysis on PDOs, we used 14 PDO lines consist of at least 6 samples for both EC- and SC- type groups, which is sufficient to demonstrate the representative results. For drug treatment experiments, we applied at least 3 biological replicates which could provide adequate statistical power in the analyses. |
| Data exclusions | No data was excluded from the analyses. |
| Replication | All experiments were independently repeated as indicated and were reliably reproduced (see Figure legends where applicable for further details). |
| Randomization | PDOs used for drug treatment experiments were allocated randomly into each treatment groups. Randomization was not relevant to other experiments. No animal study and human clinical trial was conducted in present study. |
| Blinding | Blinding was not possible as the data were acquired and analyzed by the same person that performed the experimental procedure. |

# Reporting for specific materials, systems and methods

We require information from authors about some types of materials, experimental systems and methods used in many studies. Here, indicate whether each material, system or method listed is relevant to your study. If you are not sure if a list item applies to your research, read the appropriate section before selecting a response.

## Materials & experimental systems

| n/a | Involved in the study |
|---|---|
| ☐ | ☒ Antibodies |
| ☒ | ☐ Eukaryotic cell lines |
| ☒ | ☐ Palaeontology |
| ☒ | ☐ Animals and other organisms |
| ☐ | ☒ Human research participants |
| ☒ | ☐ Clinical data |

## Methods

| n/a | Involved in the study |
|---|---|
| ☒ | ☐ ChIP-seq |
| ☒ | ☐ Flow cytometry |
| ☒ | ☐ MRI-based neuroimaging |

# Antibodies

Antibodies used

AE1/3 (1:50, Abcam, ab27988), Vimentin (1:100, CST, 5741S; 1:100, Santa Cruz, sc-6260), CK5/6 (1:50, Abcam, ab17133), p63 (1:50, Abcam, ab124762), MST1R (1:100, ATLAS, HPA008180), LMP1 (1:100, Abcam, ab78113), Ki67 (1:400, CST, 9449S); CD3e (1:100, Dako, A0452); Phospho-AKT (1:1000, CST, 4060S), AKT (1:1000, CST, 9272S), Phospho-ERK (1:1000, CST, 4370S), ERK (1:1000, CST, 4695S), Phospho-STAT3 (1:1000, CST, 9145S), STAT3 (1:1000, CST, 9139S), Cyclin A (1:200, Santa Cruz, sc-751), Cyclin B (1:200, Santa Cruz, sc-752), Phospho-Bcl-2 (1:1000, CST, 2827S), Bcl-2 (1:1000, ProteinTech, 12789-1-ap), Bax (1:1000, ProteinTech, 50599-2-lg), Cleaved Caspase 3 (1:1000, CST, 9661S), β-Actin (1:1000, Sigma, A5316)

Validation

All antibodies are from commercial sources and their validation data are available on the manufacturer's website. The appropriate dilution for all the antibodies was determined through preliminary experiments.

AE1/3 (1:50, Abcam, ab27988): https://www.abcam.com/pan-cytokeratin-antibody-ae1ae3-ab27988.html

Vimentin (1:100, CST, 5741S): https://www.cellsignal.cn/products/primary-antibodies/vimentin-d21h3-xp-rabbit-mab/5741?_=1616325160697&Ntt=5741S&tahead=true

Vimentin (1:100, Santa Cruz, sc-6260): https://www.scbt.com/p/vimentin-antibody-v9?requestFrom=search

CK5/6 (1:50, Abcam, ab17133): https://www.abcam.cn/cytokeratin-5-6-antibody-d516-b4-ab17133.html

p63 (1:50, Abcam, ab124762): https://www.abcam.cn/p63-antibody-epr5701-ab124762.html

MST1R (1:100, ATLAS, HPA008180): https://www.sigmaaldrich.com/catalog/product/sigma/hpa008180?lang=en&region=MO

LMP1 (1:100, Abcam, ab78113):https://www.abcam.cn/ebv-latent-membrane-protein-1-antibody-cs-1-4-bsa-and-azide-free-ab78113.html

Ki67 (1:400, CST, 9449S): https://www.cellsignal.cn/products/primary-antibodies/ki-67-8d5-mouse-mab/9449?_=1616325165818&Ntt=9449S&tahead=true

CD3e (1:100, Dako, A0452): https://www.agilent.com/en/product/immunohistochemistry/antibodies-controls/primary-antibodies/cd3-(concentrate)-76649#productdetails

Phospho-AKT (1:1000, CST, 4060S): https://www.cellsignal.cn/products/primary-antibodies/phospho-akt-ser473-d9e-xp-rabbit-mab/4060?_=1616325257120&Ntt=4060S&tahead=true

AKT (1:1000, CST, 9272S): https://www.cellsignal.cn/products/primary-antibodies/akt-antibody/9272?_=1616325288921&Ntt=9272S&tahead=true

Phospho-ERK (1:1000, CST, 4370S): https://www.cellsignal.cn/products/primary-antibodies/phospho-p44-42-mapk-erk1-2-thr202-tyr204-d13-14-4e-xp-rabbit-mab/4370?_=1616325303621&Ntt=4370S&tahead=true

ERK (1:1000, CST, 4695S): https://www.cellsignal.cn/products/primary-antibodies/p44-42-mapk-erk1-2-137f5-rabbit-mab/4695?_=1616325316188&Ntt=4695S&tahead=true

Phospho-STAT3 (1:1000, CST, 9145S): https://www.cellsignal.cn/products/primary-antibodies/phospho-stat3-tyr705-d3a7-xp-rabbit-mab/9145?_=1616325329548&Ntt=9145S&tahead=true

STAT3 (1:1000, CST, 9139S): https://www.cellsignal.cn/products/primary-antibodies/stat3-124h6-mouse-mab/9139?_=1616325350706&Ntt=9139S&tahead=true

Cyclin A (1:200, Santa Cruz, sc-751): https://www.scbt.com/p/cyclin-a-antibody-h-432?requestFrom=search

Cyclin B (1:200, Santa Cruz, sc-752): https://www.scbt.com/p/cyclin-b1-antibody-h-433?requestFrom=search

Phospho-Bcl-2 (1:1000, CST, 2827S): https://www.cellsignal.cn/products/primary-antibodies/phospho-bcl-2-ser70-5h2-rabbit-mab/2827?_=1616325376211&Ntt=2827S&tahead=true

Bcl-2 (1:1000, ProteinTech, 12789-1-ap): https://www.ptglab.com/Products/BCL2-Antibody-12789-1-AP.htm

Bax (1:1000, ProteinTech, 50599-2-lg): https://www.ptgcn.com/products/BAX-Antibody-50599-2-Ig.htm

Cleaved Caspase 3 (1:1000, CST, 9661S): https://www.cellsignal.cn/products/primary-antibodies/cleaved-caspase-3-asp175-antibody/9661?_=1616325397129&Ntt=9661S&tahead=true

β-Actin (1:1000, Sigma, A5316): https://www.sigmaaldrich.com/catalog/product/sigma/a5316?lang=en&region=MO

## Human research participants

Policy information about studies involving human research participants

| | |
|---|---|
| Population characteristics | Samples used for this study were collected from 106 patients who pathologically diagnosed with NPC at the Kiang Wu Hospital and the First Affiliated Hospital of Southwest Medical University. Ages of cancer patients were between 22 and 76 years (median, 48 years), 72 males and 34 females. Tumor subtype was examined by the pathology department of hospitals and further confirmed by researcher of University of Macau, with 78 NKUC, 35NKDC and 3 KSCC under WHO classification, and with 57 EC, 26 MSEC, 20 SC and 3 SCC under histological classification. Detailed clinicopathological characteristics of all NPC patients included in this study are summarized in Supplementary Table 1 and Supplementary Data 1, the prior consent to publish information of gender, age, ethnicity and associated clinical characteristics was obtained from subjects. |
| Recruitment | Participants were recruited by participating physicians at the Kiang Wu Hospital and the First Affiliated Hospital of Southwest Medical University. Prior patient written consent was obtained from donors with informing the use for genomics sequencing, organoid culture, drug test, publications and associated scientific studies. Patients were not selected for treatment and samples of blood or tumor tissues were collected during standard clinical procedures. Results of this study for individual patients were not available for participating physicians or patients during sample collection, which excludes potential bias during patient recruitment. |
| Ethics oversight | This study was assessed and approved by the ethics committees of University of Macau, Kiang Wu Hospital and First Affiliated Hospital of Southwest Medical University. |

Note that full information on the approval of the study protocol must also be provided in the manuscript.
