# Supplementary material for: Molecular landscape and subtype-specific therapeutic response of nasopharyngeal carcinoma revealed by integrative pharmacogenomics
Source: Nat Commun. 2021 May 24;12:3046. doi: 10.1038/s41467-021-23379-3 (PMC8144567; doi:10.1038/s41467-021-23379-3)
Supplement: Supplementary file 4 — Description of Additional Supplementary Files [file 41467_2021_23379_MOESM4_ESM.pdf]

Description of Additional Supplementary Files

Supplementary Data 1
Description: Detailed clinicopathological characteristics of NPC patients (n=106)

Supplementary Data 2
Description: H&E staining images of 47 tumors for bulk sequencing

Supplementary Data 3
Description: Somatic mutational events identified by WES in this cohort

Supplementary Data 4
Description: Sanger sequencing validation of candidate mutations

Supplementary Data 5
Description: 48-drug screening results (42 PDOs)

Supplementary Data 6
Description: First-line CRT screening results (20 PDOs)

Supplementary Data 7
Description: 48-CRT screening results (8 PDOs)

Supplementary Data 8
Description: Gene cohorts with subtype-differentiated expressions

Supplementary Data 9
Description: Sanger sequencing validation for CRISPR-Cas9 KO experiment

Supplementary Data 10
Description: Formulation of tissue dissociation buffer and organoid culture medium

Supplementary Data 11
Description: Summary of sequencing coverages

Supplementary Data 12
Description: Drug library used for high-throughput screening (146 drugs)

Supplementary Data 13

Description: sgRNA oligos used for CRISPR-Cas9 KO experiment
